# Breast Tumor Cell Invasion and Pro-Invasive Activity of Cancer-Associated Fibroblasts Co-Targeted by Novel Urokinase-Derived Decapeptides

**DOI:** 10.3390/cancers12092404

**Published:** 2020-08-24

**Authors:** Stefania Belli, Paola Franco, Francesca Iommelli, Anna De Vincenzo, Diego Brancaccio, Marialucia Telesca, Francesco Merlino, Ettore Novellino, Marie Ranson, Silvana Del Vecchio, Paolo Grieco, Alfonso Carotenuto, Maria Patrizia Stoppelli

**Affiliations:** 1 Institute of Genetics and Biophysics “Adriano Buzzati Traverso”, National Research Council, 80131 Naples, Italy; stefania.bell21@gmail.com (S.B.); paola.franco@igb.cnr.it (P.F.); annacdevincenzo@gmail.com (A.D.V.); lucia.telesca@igb.cnr.it (M.T.); 2 Institute of Biostructure and Bioimaging, CNR, 80131 Naples, Italy; francesca.iommelli@ibb.cnr.it; 3 Department of Pharmacy, University of Naples “Federico II”, 80131 Naples, Italy; diego.brancaccio@unina.it (D.B.); francesco.merlino@unina.it (F.M.); ettore.novellino@unina.it (E.N.); paolo.grieco@unina.it (P.G.); 4 Illawarra Health and Medical Research Institute, Wollongong, NSW 2522, Australia; mranson@uow.edu.au; 5 School of Chemistry and Molecular Biosciences, University of Wollongong, Wollongong, NSW 2522, Australia; 6 Department of Advanced Biomedical Sciences, University of Naples “Federico II”, 80131 Naples, Italy; delvecc@unina.it

**Keywords:** tumor microenvironment, metastatic dissemination, breast cancer cell invasion, αv integrin, cancer-associated fibroblasts

## Abstract

Among peritumoral cells, cancer-associated fibroblasts (CAFs) are major facilitators of tumor progression. This study describes the effects of two urokinase-derived, novel decapeptides, denoted as Pep 1 and its cyclic derivative Pep 2. In a mouse model of tumor dissemination, using HT1080 fibrosarcoma cells, Pep 2 reduced the number and size of lung metastases. Specific binding of fluoresceinated Pep 2 to HT1080 and telomerase immortalised fibroblasts (TIF) cell surfaces was enhanced by αv overexpression or abolished by excess vitronectin, anti-αv antibodies or silencing of *ITGAV* αv gene, identifying αv-integrin as the Pep 2 molecular target. In 3D-organotypic assays, peptide-exposed TIFs and primary CAFs from breast carcinoma patients both exhibited a markedly reduced pro-invasive ability of either HT1080 fibrosarcoma or MDA-MB-231 mammary carcinoma cells, respectively. Furthermore, TIFs, either exposed to Pep 2, or silenced for αv integrin, were impaired in their ability to chemoattract cancer cells and to contract collagen matrices, exhibiting reduced α-smooth muscle actin (α-SMA) levels. Finally, peptide exposure of αv-expressing primary CAFs led to the downregulation of α-SMA protein and to a dramatic reduction of their pro-invasive capability. In conclusion, the ability of the novel decapeptides to interfere with tumor cell invasion directly and through the down-modulation of CAF phenotype suggests their use as lead compounds for co-targeting anti-cancer strategies.

## 1. Introduction

In the past solid tumors were regarded as relatively homogeneous groups of hyperproliferating cells with the ability to invade neighboring tissues and, possibly, metastasize. More recently, a large body of evidence has convincingly revealed that tumors are complex organs composed of multiple cell types and extracellular matrix (ECM) [1,2]. Intensive studies on solid mammary, lung, intestinal and prostatic cancer have described a surrounding tumor microenvironment (TME), that includes infiltrated immune cells (T cells and macrophages), endothelial cells, pericytes, adipocytes and cancer-associated fibroblasts (CAFs). During the development of solid tumors, all these components work synergistically, ultimately becoming supportive of cancer progression, dissemination and resistance to chemotherapeutics [3]. Among TME cells, CAFs, also defined as myofibroblasts, are the most abundant cell type and orchestrate paracrine pro-tumorigenic signaling with adjacent tumor cells, thus accelerating tumor progression [4]. Although in physiological conditions, tissue fibroblasts are characterised by negligible transcriptional and metabolic activity, they may be activated during tumorigenesis, and acquire a wide spectrum of new abilities that sustain the establishment of solid tumors. Whether CAF phenotype in TME is reversible, and to what extent, is still debated. CAFs are identified by the overexpression of several activation markers, like α-smooth muscle actin (α-SMA), and fibroblasts activation protein (FAP), associated to the loss of caveolin-1 (CAV-1) [4]. Recruitment and activation of peritumoral fibroblasts are mediated by growth factors secreted by cancer epithelial and by several TME cell types, such as transforming growth factor-β (TGF-β), platelets-derived growth factor (PDGF) and fibroblast growth factor-2 (FGF-2) [5]. Recent evidence highlights the relevance of IGF-1 and IGF-2 (Insulin-like growth factors 1 and 2), secreted by mammary tumor epithelial cells and inducing the recruitment and activation of patient-derived primary CAFs through the IGF-1R [6].

Mechanistically, CAFs can contribute to cancer progression via integrin-linked mechanisms, through the generation of pro-migratory tracks favoring cancer cell invasion in the stromal ECM, and through αvβ3 integrin-dependent matrix reorganization and fibronectin assembly [7,8]. Integrins are heterodimeric cell surface receptors, which enable adhesion, proliferation, and migration of cells by recognizing binding motifs in ECM proteins. Notably, a subset of integrin receptors is overexpressed on the surface of tumor and stromal cells, with a high impact on tumor progression [9,10]. Of particular interest, the αv integrins, particularly αvβ3, αvβ6, and αvβ8 integrin, reported to be expressed in several solid tumors [11]. Among the most promising anti-αv integrins drugs, abituzumab, intetumumab, two pan-αv integrin antibodies and the cyclic pentapeptide cilengitide, that specifically inhibits αvβ3 and αvβ5. Despite their pre-clinical efficacy, most of these treatments failed their primary endpoints in phase 2 and phase 3 trials [9]. However, alternative approaches may be considered, including their inhibition in non-neoplastic peritumoral cells, as they still remain valid therapeutic targets [12].

This work is focused on the design, conformational and functional analysis of novel decapeptides endowed with the ability to prevent tumor migration and invasion. Previous work indicates that the serine protease urokinase (uPA) has a catalytically-independent motogen activity that resides in its amino-terminal growth factor domain (GFD, residues 1–49) and in its connecting peptide region (CP, residues 132–158) [13]. The CP region binds to αv integrin, bridging uPAR and the αvβ5 [14]. Most of the chemotactic activity of CP-derived peptide uPA-(135–158), named CPp, (Table 1) is retained by its C-terminal segment uPA-(144–158). In contrast, the N-terminal, uPA-(135–143) peptide, is inhibitory [15]. Furthermore, following phosphorylation of Ser138/139, full uPA acquires remarkable anti-migratory properties, which are retained by the Glu-substituted forms S138E/S303E [16,17]. Previous conformational analysis of the CP-derived, anti-migratory peptides, showed the occurrence of a turn structure around the 140–143 segment, which brings its flanking N- and C-terminal regions close to each other [15]. These findings were instrumental and suggested the development of two novel peptides whose sequence corresponds to the N-terminal region of CPp, incorporates the S138E substitution, and allows the E138-K145 side chain to side chain contact, thus stabilizing the putative bioactive conformation. In this study, we show that the two novel decapeptides, based on these characteristics, denoted as Pep 1 and Pep 2 (Table 1) prevented mouse lung metastases. They also inhibited migration and invasion of HT1080 fibrosarcoma and MDA-MB-231 breast carcinoma cells through the αv-integrin subunit. Furthermore, the novel peptides induced a partial reversion of the CAF phenotype and markedly reduced the pro-invasive ability of peritumoral CAFs from breast cancer patients in combination with MDA-MB-231 mammary adenocarcinoma cells.

## 2. Results

### 2.1. Peptide Design

To identify new molecules of pharmacological interest, one possibility is to reprogram and shorten sub-domains of relevant proteins with modular molecular structure. In the present study, two novel decapeptides derived from the CP region of human uPA, namely Pep 1 and Pep 2 (also denoted as uPAcyclin) are presented (Table 1).

**Figure 1:**
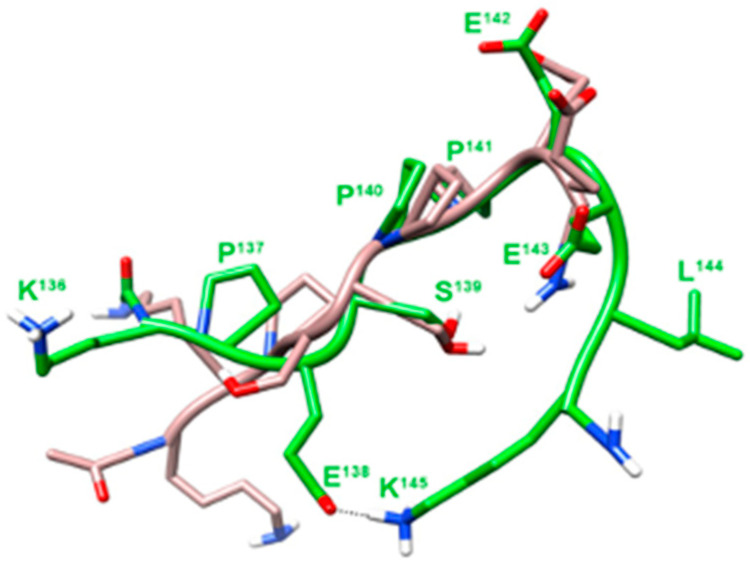
Superposition of the lowest energy conformer of uPA-(135–143) and Pep 1. Structures of uPA-(135–143) (grey) and Pep 1 (green) were superimposed using the backbone heavy atoms. Heavy atoms are shown with different colors (carbon, grey or green; nitrogen, blue; oxygen, red; hydrogen, white). For the sake of clarity, not all hydrogen atoms are depicted. Heavy backbone atoms are shown in ribbon format.

Conformational analysis of previously published anti-migratory peptides, corresponding to the N-terminal region of CP, denoted [138E]uPA-(135–158), and uPA-(135–143), demonstrated the existence of a β-turned structure, encompassing residues 140–143 (Figure 1). This secondary structure was not detected in the uPA-(135–158) or CPp peptide, endowed with a clear-cut pro-migratory activity [15]. These findings suggested that the folding induced by the β-turn could shorten the distance between the N- and C-terminal flanking regions. In the linear Pep 1, two further C-terminal residues were included in the [138E]uPA-(135–143) and the N-terminal Lys135 was omitted. In the resulting Pep 1, NMR analysis confirmed the proximity of the side chains of Glu138 and Lys145 residues (Figure 1). This observation prompted us to design Pep 2, in which the side chains of Glu138 and Lys145 were covalently linked by an amide bond.

### 2.2. Conformational Analysis

Conformational analysis of Pep 1 and Pep 2 was carried out by Nuclear Magnetic Resonance (NMR) in water solution. Pep 1 showed a β-turn around the Pro142-Glu143, as evinced by a diagnostic NOE between Hα of residue Pro142 and HN of Glu144, with flanking regions in extended conformation. Structure calculation based on NMR derived constraints disclosed a type I β-turn flanked by two β-strand structures. Many conformers (about 60%) also displayed a salt bridge between Glu138 and Lys145 side chains (Figure 1). In Pep 2 NMR spectra, most features resemble those of Pep 1. However, about half of the peptide molecules exhibited the Ser139-Pro140 amide bond in cis configuration. Unfortunately, most signals arising from the trans and cis sub-populations are overlapping, thus preventing us from obtaining the necessary NOEs for structure calculation.

### 2.3. Inhibition of Malignant Cell Lung Colonization In Vivo by Pep 2

Since intra-molecular cyclisation may increase peptide stability and permeability, the anti-tumoral properties of Pep 2 were first tested in vivo. To this end, HT1080, a human fibrosarcoma cell line metastasising to the lungs in nude mice, were exposed to 1 µM Pep 2 for 1 h and injected into the tail vein of 15 mice. Of these, 5 mice received Pep 2 at 0.068 mg/day (3.4 mg/kg/day) every day, and 5 mice received 0.3 mg/day (15 mg/kg/day) for 10 days every day and then once a week, whereas 5 control mice received injections of vehicle only.

**Figure 2:**
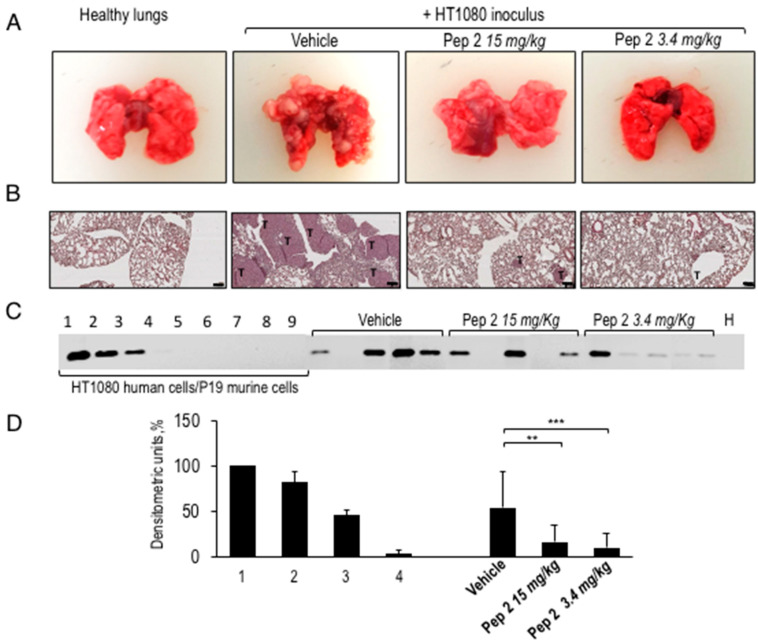
Macroscopic view of whole lungs and hematoxylin and eosin (H&E)-stained lung sections from Pep 2-treated mice. (**A**) Representative images of lungs surgically removed from healthy animals or from animals intravenously injected with HT1080 cells: of these, 5 were untreated (vehicle), 5 were treated with Pep 2 either at 15 mg/kg or at 3.4 mg/kg. (**B**) H&E-stained of the corresponding representative lung sections with the neoplastic areas marked with T (4× magnification, Scale bar 100 µm. (**C**) 50 ng of total genomic DNA extracted from lung samples from each vehicle, or Pep 2-treated mice were used as template for semi-quantitative PCR reactions and compared to the healthy lungs (H). The amplification products are separated on 1% agarose gels. A standard curve was included in every run, generated by mixing 10^7^, 10^6^,10^5^,10^4^,10^3^,10^2^,10^1^,1 HT1080 human cells with 1 to 10^7^ murine P19 cells (Standard 1 to 9). The uncropped image for agarose gel can be found in Figure S5. (**D**) The densitometric analysis of PCR amplification products, accomplished by acquiring images and quantifying them through ImageJ software (NIH, Bethesda, MD, USA), was performed on three independent gels. The signal from the 1 × 10^7^ HT1080 cells sample was considered as 100% and standards 2, 3, 4 and 5 were graded relative to that (** *p* < 0.005; *** *p* < 0.001, Student’s *t*-test).

After 28 days, mice were sacrificed, lungs were surgically removed and compared with lungs from five healthy mice. Figure 2A shows the macroscopic view of whole lungs from healthy, untreated and peptide-treated animals. Morphometric analysis of lung metastatic foci revealed a mean neoplastic area significantly lower in Pep 2-treated compared to untreated mice (Figure 2A). Accordingly, histological analysis showed that intra-parenchymal and sub-pleural lung metastatic foci in untreated mice dramatically decrease when mice were treated with Pep 2, in both the treatment schedules (Figure 2B).

To obtain a quantitative measure of the treatment efficiency, one lung of each animal was subjected to a total genomic DNA extraction to quantify the amount of Alu sequences in murine lung samples amplified. The PCR amplification products were separated on agarose gel (Figure 2C). The number of human cells in murine lung samples was calculated by comparing the obtained PCR amplification signals with a standard curve, included in every run, generated by mixing decreasing numbers of HT1080 human cells with increasing numbers of mouse P19 embryonic cells, and keeping 10^7^ as total cell number. DNA from healthy murine lung sample was included as a control (H). In Figure 2D, the ImageJ analysis reveals that the amplification signal of Alu sequences from lungs of mice treated with 15 mg/kg peptide discontinuously or with 3.4 mg/kg every day, was three to four-fold lower, respectively, compared to lungs of untreated mice. Animals were daily monitored and neither severe signs of toxicity nor dysfunction of normal organs or weight loss higher than 10% were observed after Pep 2 peptide treatment. These data show that Pep 2 has a clear-cut anti-metastatic activity, preventing malignant cell lung colonization at micromolar concentrations. Remarkably, the 3.4 mg/kg every day lower dosage is equally effective than the discontinuous treatment at 15 mg/kg.

### 2.4. Inhibition of Fibrosarcoma Invasion in 3D-Organotypic Assay by Pep 1 or Pep 2 Decapeptides

**Figure 3:**
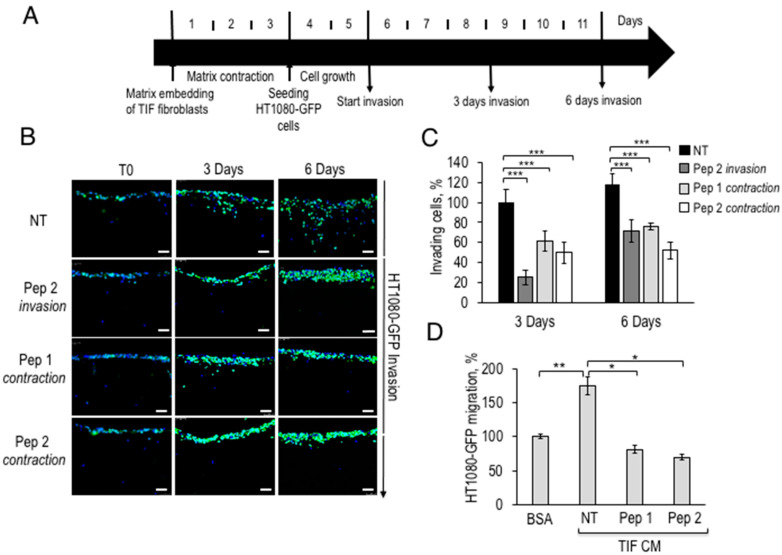
Fibrosarcoma cell invasion in the presence of fibroblasts pre-exposed to Pep 1 or Pep 2 in 3D-Organotypic assays. (**A**) Time scale representation of the 3D-organotypic invasion assay. 5 × 10^4^ TIF fibroblasts/sample were first embedded into a neutralized collagen I solution and incubated for 3 days. Then 1 × 10^5^ HT1080-GFP cells were seeded on the matrices, grown for 2 days (T0) and let to invade for 3 or 6 days. Matrices were then processed as described in Section 4.14. (**B**) Representative images of matrices sections after DAPI staining, 20× magnification, Scale bar, 50 µm. Fibroblasts were processed as described in (**A**), in the absence of Pep 2 (NT). Duplicate samples were exposed to 100 nM Pep 2 during HT1080-GFP invasion (Pep 2 invasion) or to 100 nM Pep 1 or Pep 2 during matrix contraction (Pep 1 contraction, Pep 2 contraction) and then peptides were removed and the matrices washed twice with growth medium. During contraction or invasion peptides were added to matrices every day. (**C**) The number of invading cells was quantified by densitometric analysis of images shown in B, with ImageJ software, the number of HT1080-GFP invading cells on untreated matrices was normalized to T0 and the 3 days sample was considered as 100%. (**D**) 5 × 10^4^ TIF fibroblasts were seeded in DMEM-10% FBS in 6 well plates for 24 h, serum-starved for 6 h and incubated for 24 h in presence or in the absence of 100 nM Pep 1 or Pep 2 peptides in DMEM-10%FBS. Peptides were then removed and, after two PBS 1× washes, cells were incubated in serum-free medium for 24 h. Conditioned media (CM) from TIFs exposed to Pep 1 or Pep 2 were collected and employed as a chemoattractants for H1080-GFP cells in Boyden chamber migration assays. In each sample, 2 × 10^4^ HT1080-GFP cells were tested either toward DMEM-0.1% BSA (basal migration) or toward TIFs CM. Basal migration was taken as 100% and all values were calculated relative to that. * *p* < 0.05; ** *p* < 0.005; *** *p* < 0.001, Student’s *t*-test.

Among the peritumoral cell types, myofibroblasts are the most abundant, and active to remodel ECM, induce angiogenesis, proliferation, invasion, and resistance to cell death [4]. The 3D organotypic invasion assays with matrix-embedded fibroblasts recapitulate TME and may provide information about the cells responding to peptide inhibition. To this end, telomerase immortalised fibroblasts or TIF, with a marked ability to contract matrices and expressing α-SMA, were co-cultured with HT1080 human fibrosarcoma cells to monitor invasion of the underlying matrix in the presence or in the absence of Pep 1 or Pep 2. During the initial three days, in the “matrix contraction” phase, collagen-embedded TIF fibroblasts undergo further matrix deposition to generate a stiff collagen disk. Afterwards, peptides were accurately removed and GFP-expressing HT1080 cells, were seeded on top of matrices and left to grow for two days and then to invade either for 3 or for 6 days (Figure 3A). Pep 1 or Pep 2 were either included only in the contraction phase (contraction), thus exposing only TIF fibroblasts, and then removed, or applied throughout the experiment (invasion).

As summarized in the legend to Figure 3A, after 3 or 6 days of invasion, matrices fixed, sectioned, and stained with DAPI and representative images were captured with a fluorescence microscope (Figure 3B). As shown in Figure 3B, Pep 2, included during the invasion phase, reduced HT1080-GFP invasion by 80% after 3 days of invasion and by over 40%, after 6 days. The number of invading cells was quantified by ImageJ software and unexposed cells were taken as 100% (Figure 3C). If TIFs were exposed to Pep 1 or Pep 2 only during the matrix contraction phase, and subsequently removed during the invasion phase, tumor invasion was impaired by 50–60% at 3 and 6 h (Pep 1 and Pep 2 contraction, Figure 3C). The latter findings suggest that Pep 2-exposed TIF matrices are not fully permissive to HT1080 invasion. It has to be noticed that TIFs pre-exposure to Pep2 leads to a 40% decrease of HT1080 invasion, whereas Pep2-treatment of both, HT1080 and TIFs, leads to an 80% inhibition, after 3 days. In separate experiments, we have determined that TIFs express about 40% of the αv integrin protein levels expressed by HT1080 cells (Franco P. and Stoppelli M.P., Signaling effects of Pep 2 via αv-Integrin, unpublished). The data overall confirm a direct effect of Pep 2 on HT1080 invasion and suggest a partial contribution by TIFs to the inhibition of tumor cell invasion.

Whether also mouse CAFs are Pep 2-sensitive remains to be determined. To investigate whether the impaired invasion is due to reduced secretion of motogen factors by peptide-treated fibroblasts, serum-free CM from Pep 1 or Pep 2-exposed TIF fibroblasts was tested as chemoattractant for HT1080-GFP cells. Figure 3D shows a significant mobilization effect of HT1080-GFP by CM from untreated TIF in directional migration assays in Boyden chambers. In contrast, CM from peptide-treated fibroblasts lacked the ability to chemoattract HT1080-GFP cells, actually exhibiting a weak inhibitory effect of fibrosarcoma cell basal migration. To investigate the indirect mechanisms modulating tumor invasion, TIFs were exposed to metallo-protease (MMP) inhibitors. GM6001-treated fibroblasts exhibit reduced matrix contraction and pro-invasive abilities, suggesting that MMPs are relevant to these fibroblast properties (Franco P. and Stoppelli M.P., Signaling effects of Pep 2 via αv-Integrin, unpublished). If the novel peptides interfere with the secretion of motogen factors and/or with MMP synthesis/secretion remains to be determined.

### 2.5. Inhibition of HT1080 Cell Invasion by αv Integrin Receptor Interaction with Pep 1 or Pep 2

**Figure 4:**
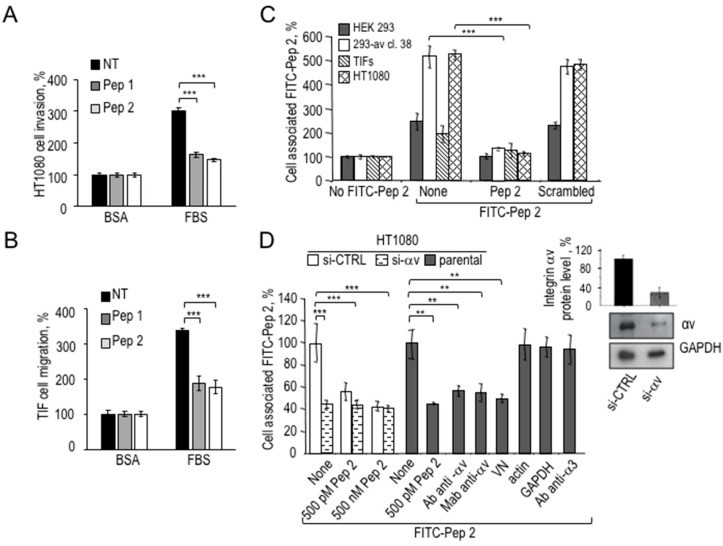
Pep 1 and Pep 2 peptides inhibit HT1080 invasion by binding to αv integrin subunit. (**A**) 2 × 10^4^ HT1080 cells/sample or (**B**) 5 × 10^4^/sample TIF fibroblasts were pre-incubated with or without 100 nM Pep 1 or Pep 2 for 1 h at 37 °C and then assayed in Boyden chambers at 37 °C, either for FBS-dependent invasion on matrigel for 5 h (**A**) or for FBS-dependent migration for 3 h (**B**). Data are expressed as in the legend to Figure 3D (*** *p* < 0.001, Student’s *t*-test). (**C**) 2 × 10^6^ HEK-293 cells, or HEK-293-αv-38, TIF or HT1080 cells were pre-incubated for 30 min at 4 °C with an excess of unlabeled Pep 2 or scrambled peptides (500 nM) and exposed to 50 nM FITC-Pep 2 for 2 h at 4 °C. Cell surface-associated fluorescence, as percentage of the samples with no FITC-Pep 2, is shown. Data represent a mean of three independent experiments performed in duplicate (*** *p* < 0.001, Student’s *t*-test). (**D**) HT1080 cells were transiently transfected with si-control (si-CTRL) or si-RNA targeting αv integrin mRNA (si-αv). The efficiency of silencing was assessed by Western Blot using polyclonal anti-αv antibody or monoclonal anti-GAPDH as loading control and quantified by Image J (inset). The whole blot image can be found in Figure S6. After 48 h, 2 × 10^6^ cells/sample were analysed for FITC-Pep 2 specific binding, as described in panel (**C**). As indicated, HT1080 cells were pre-treated with anti-αv polyclonal (Ab), or monoclonal antibody (Mab) or anti-actin polyclonal or anti-GAPDH monoclonal antibodies or with Pep 2 or 200 nM vitronectin (VN) for 1 h at 37 °C and analyzed for FITC-Pep 2 specific binding. Data represent a mean of three independent experiments performed in duplicate (** *p* < 0.005; *** *p* < 0.001, Student’s *t*-test).

To further dissect HT1080 and TIF cell responses to Pep 1 and Pep 2, their ability to prevent directional cell migration and invasion was tested. Unlike the scrambled peptide (Table 1), Pep 1, and Pep 2 reduce FBS-dependent migration of HT1080 cells in a dose-dependent manner, IC_50_ being around 10^−10^ M (Figure S1A). Also, HT1080 matrix invasion in Boyden chamber assays was strongly inhibited by Pep 1 and Pep 2 (Figure 4A). Interestingly, TIF migration is indeed prevented by Pep 1 or Pep 2, indicating a clear-cut response to both peptides (Figure 4B).

We also assessed the effects of Pep 2 on HT1080 migration by a scratch would healing assay. In untreated controls, the wound appeared closed after 24 h, whereas Pep 2 reduced HT1080 wound closure by 20% after 12 h and by 60–70% after 24 h (Figure S1B). As shown in Figure S2A, TIF fibroblasts were monitored for 24 h under the same conditions and quantitative data are reported in Figure S2B. Again, Pep 2 could reduce the speed of wound closure at any time point, the process being slowed by 20–70% after 12 and 24 h, respectively. In conclusion, cell exposure to Pep 2 leads to a significant inhibition of HT1080 fibrosarcoma and TIF fibroblasts random migration.

To investigate the specific interaction of the novel peptides with the surface of cells employed in this study, binding assays with FITC-Pep 2 peptide were performed. Previously published peptides, derived from the uPA CP region, were characterised by a high affinity binding to the αv integrin subunit [15]. Therefore, human embryonic kidney HEK-293 and the relative αv- stably overexpressing counterpart HEK-293-αv, were tested to check for increased binding of FITC-Pep 2, together with TIF fibroblasts and HT1080 fibrosarcoma cells, used in this study. All cells were pre-incubated with an excess unlabeled Pep 2 or scrambled Pep 2 peptides and then exposed to FITC-Pep 2. Cell surface-associated fluorescence was measured, the reference 100% being cells unexposed to FITC-Pep 2 (Figure 4C). Unlike the scrambled peptide, FITC-Pep 2 specifically associated with the surface of all cells examined. In particular, HEK-293-αv exhibited a 3–4-fold increased binding, as compared to parental HEK-293 cells indicating that Pep 2 binding increases in cells overexpressing αv integrin. Although to a different extent, FITC-Pep 2 specifically associates to HT1080 fibrosarcoma cells and TIF fibroblasts. To further confirm the αv integrin receptor as a molecular target of Pep 2, the expression of αv subunit was silenced in HT1080 cells, subsequently tested for the extent of FITC-Pep 2 binding. A 70% silencing efficiency, compared to cells bearing control siRNA, was revealed by Western Blot analysis and relative quantification (Figure 4D, inset). As shown in Figure 4D, HT1080 transfected with si-CTRL specifically bound to FITC-Pep 2, unless pre-incubated with 500 nM Pep 2. In contrast, αv-silenced HT1080 cells fail to specifically bind FITC-Pep 2. Furthermore, cell pre-treatment with monoclonal or polyclonal blocking antibodies to αv integrin or purified vitronectin for 1 h at 37 °C abolished FITC-Pep 2 specific binding. Conversely, antibodies against α3 integrin or polyclonal anti-actin or monoclonal anti-GAPDH were ineffective, confirming the specificity of Pep 2 binding to the αv integrin subunit.

### 2.6. Partial Reversion of CAF-Like Phenotype in TIF Fibroblasts Exposed to Pep 1 or Pep 2

As shown by the organotypic assays, the two αv integrin binding novel peptides, may counteract the pro-invasive ability of TIF fibroblasts through the inhibition of fibroblast-secreted motogen factors (Figure 3). In light of the crucial role of ECM in regulating neoplastic progression and providing biochemical cues and mechanical scaffolding to cell invasion, the possibility that peptide-exposure of TIFs leads to an altered non-permissive, matrix deposition, was examined.

**Figure 5:**
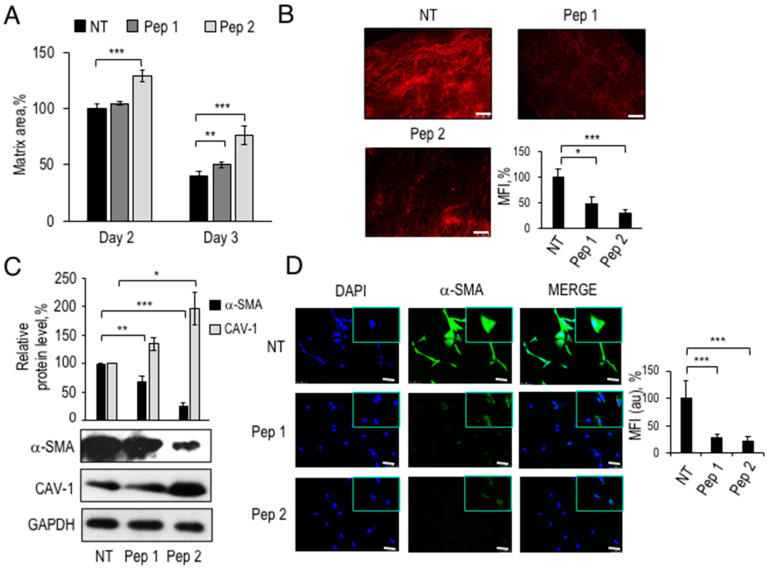
Modulation of CAF-like phenotype by Pep 1 and Pep 2. (**A**) Contraction assay of 5 × 10^4^ TIF fibroblasts, pre-treated for 1 h at 37 °C with diluents (NT) or 100 nM Pep 1 or Pep 2 in DMEM-10% FBS. TIF cells were mixed with a collagen I solution, in absence or presence of 100 nM Pep 1 or Pep 2. Peptides were added every day for 3 days. Matrix area contracted by untreated fibroblasts at day 2 was considered as 100% and the other samples considered relative to that. (**B**) Representative images of sections stained with 594-PSR picrosirius red of matrices obtained in (**A**), 20× magnification, Scale bar 50 µm. Quantitation of fluorescence signal was performed with ImageJ software. The mean fluorescence intensity (MFI) of untreated matrices was considered as 100%. (**C**) Western Blot analysis of TIF cell lysates with polyclonal anti-α-SMA, polyclonal anti-CAV-1 and anti-GAPDH antibodies. 5 × 10^4^ TIF were seeded in 6 well plates in DMEM-10% FBS, grown for 24 h, serum-starved for 6 h, and then exposed to 100 nM Pep 1 or Pep 2 peptides in the presence of DMEM-10% FBS. Peptides were re-added to the medium after 24 h and total cellular lysates were prepared after 48 h. Densitometric analysis of Western Blot is shown on top. The whole blot image can be found in Figure S7. (**D**) For immunofluorescence analysis, 2 × 10^4^ fibroblasts were seeded in DMEM-10% FBS on cover slips for 24 h, serum-starved for 6 h and treated with Pep 1 or Pep 2 in DMEM-10% FBS. Peptides were re-added every day to the medium for 72 h. Cells were fixed in 4% PFA and stained with DAPI or anti-α-SMA antibody. Immunofluorescence images (20× Magnification and 63× in the insets) are representative of three separate experiments. The intra-cellular fluorescence signal following staining with anti-α-SMA antibody was measured by ImageJ software and the fluorescence signal of untreated fibroblasts was considered as 100%. * *p* < 0.05; ** *p* < 0.005; *** *p* < 0.001, Student’s *t*-test.

To monitor the deposition of the collagen matrix by fibroblasts, TIFs, pre-treated with Pep 1 or Pep 2, were included in a collagen I neutralized solution, and the area of resultant matrices measured after 2 and 3 days (Figure 5A). Relative to the area of the matrix reorganized by untreated TIFs at Day 2 (100% control), cells pre-exposure to Pep 1 led to a slight, but significant, increase in matrix area, whereas Pep 2 led to 30–50% larger matrices, after 2 and 3 days, respectively. To test whether collagen I fibers density was affected by the peptides, matrices were subjected to Picrosirius red staining (Figure 5B). This procedure revealed a 50–70% decrease in fibers density after treatment with Pep 1 and Pep 2, respectively, suggesting the impairment of matrix contraction and collagen deposition by fibroblasts exposed to 100 nM peptides. However, this possibility requires a validation with other ECM remodeling signatures.

These findings support the possibility that peptide-treated TIF fibroblasts become non-permissive to tumor cell invasion not only because of decreased motogen factors secretion, but also because of impaired matrix deposition. Evidence from the literature shows that primary fibroblasts switch to a myofibroblast-like phenotype, under common culture conditions, even in the absence of FBS [18]. Increased ECM deposition, soluble factors secretion and migratory behavior characterize fibroblasts with a CAF-like phenotype. Among the characteristics of fibroblast activation, are an increased α-SMA and decreased CAV-1 protein levels. Thus, we evaluated the expression level of two relevant CAF markers in peptide-exposed and unexposed TIFs. As shown in Figure 5C, TIFs express a basal level of α-SMA, indicating a partial CAF-like phenotype. After 48 h preincubation with either peptide, Pep 1 or Pep 2 downregulated α-SMA protein levels by 30% and 80%, respectively. In contrast, CAV-1 protein levels reached 120% in Pep 1-treated fibroblasts and nearly 200% in Pep 2-treated fibroblasts, suggesting that both peptides induce a partial deactivation of TIFs.

The decrease in α-SMA protein levels was assessed also by immunofluorescence with polyclonal anti-α-SMA antibody after 72 h of treatment with either Pep 1 or Pep 2 (Figure 5D). In treated cells, not only the number of α-SMA-positive cells decreases, but also a general decrease in intra-cellular fluorescence signal was observed, compared to control cells. Histograms in Figure 5D show a 70% and an 80% decrease in α-SMA fluorescence signal in Pep 1-treated and Pep 2-treated TIF, respectively, compared to untreated TIF taken as 100%.

In conclusion, the reduced ability to secrete motogen factors, to efficiently contract collagen matrices, together with the increased CAV-1 and decreased α-SMA expression markers, indicates that Pep 1 and Pep 2 decapeptides induce a partial reversion of the CAF-like phenotype of TIFs, resulting in an overall impairment of fibrosarcoma cells invasion in 3D-organotypic co-cultures.

### 2.7. Proliferation and Apoptosis of TIF and HT1080-GFP Cells Unaffected by Pep 2

The effects of Pep 2 on cell proliferation and apoptosis of HT1080-GFP and TIF cells were assessed. As shown in Figure S3A,C, both cell lines did not proliferate in culture, in the absence of FBS. In particular, the growth curves of HT1080-GFP, in the presence or absence of Pep 2, are perfectly superimposable, showing no changes of proliferation rate (Figure S3A). Pep 2-treated TIF cells showed a slight but not significant decrease in the proliferation rate after 24 and 48 h (Figure S3C). The possibility that Pep 2 may be pro-apototic was investigated by a caspase 3/7 apoptosis luminometric assay (Figure S3B,D) on TIF and HT1080-GFP cells exposed for 24 and 48 h to Pep 2. In both cases, serum-deprived cells were taken as 100% and apoptosis in samples with FBS was calculated relative to that. FBS strongly reduced the extent of apoptosis and the inclusion of Pep 2 did not further enhance the effects of serum.

### 2.8. Inhibition of CAF-Like Phenotype and Pro-Invasive Activity of αv-Silenced TIF Fibroblasts

**Figure 6:**
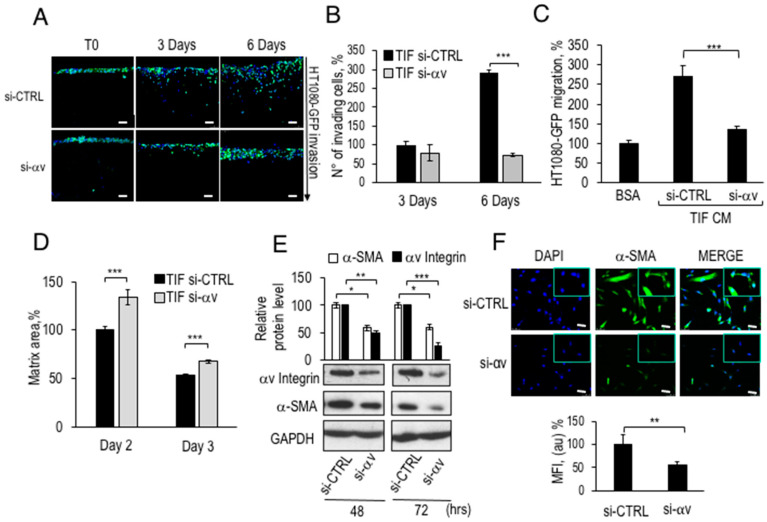
Decreased pro-invasive ability, matrix contraction capacity, and α-SMA protein levels in αv-silenced TIF fibroblasts. (**A**) Organotypic assay conducted with 5 × 10^4^ TIF fibroblasts, transfected with siRNA CTRL or si-αv, combined with a collagen I solution. 1 × 10^5^ HT1080-GFP cells were seeded on top of these matrices, incubated for 2 days (T0) and let to invade for 3 or 6 days. Matrices were processed as described in the legend to Figure 3. Scale bar 50 µm. (**B**) The number of invading HT1080-GFP cells in (**A**) was quantified, normalized to T0 and reported as percentage of si-CTRL fibroblasts after 3 days, considered as 100%. (**C**) 5 × 10^4^ TIF fibroblasts, transfected with si-CTRL or si-αv, were grown for 48 h in DMEM-10% FBS and serum-starved for 24 h. CM were collected and employed as chemoattractants for HT1080-GFP in Boyden chamber assays as described in the legend of Figure 3D. (**D**) Contraction assay carried out with 5 × 10^4^ TIF fibroblasts transfected with siRNA CTRL or siRNA to integrin αv and, after 24 h, mixed with the collagen I solution. The reduction of matrix diameter was monitored for 3 days. The area of matrices with TIF fibroblasts transfected with si-CTRL at day 2 was taken as 100%. (**E**) Western blot of total lysates from fibroblasts transfected with siRNA CTRL or siRNA to αv integrin were collected and subjected to quantitation of α-SMA and αv protein levels using GAPDH as a reference. The whole blot image can be found in Figure S8. (**F**) 2 × 10^4^ fibroblasts were seeded in DMEM-10% FBS on cover slips in 6 well plates for 24 h and silenced for integrin αv expression by RNA interference. After 72 h, cells were fixed and stained with DAPI or anti-α-SMA antibody by immunofluorescence (20× magnification and 63× in the inset). Scale bar 50 µm. The MFI following staining with anti-α-SMA antibody was quantified and reported as indicated in the legend to Figure 5D. * *p* < 0.05; ** *p* < 0.005; *** *p* < 0.001, Student’s *t*-test.

As shown in Figure 4, αv integrin subunit is the cellular target of Pep 2, and therefore its silencing should result in outcomes similar to those observed in peptide-treated fibroblasts. To evaluate whether αv-depleted TIFs, had similar characteristics to those of Pep 1- or Pep 2-exposed fibroblasts, αv silenced-TIF cells were embedded in collagen matrices and subjected to 3D-organotypic assays with HT1080-GFP cells. As shown in Figure 6A, the invasion of fibrosarcoma cells was dramatically impaired in matrices remodeled by TIF fibroblasts silenced for αv integrin expression. Figure 6B shows that the number of invading cells, into matrices with αv-silenced fibroblasts, was reduced to baseline levels after 6 days of invasion, compared to TIF bearing the si-CTRL. In separate experiments we have determined that αv silencing affects TIFs proliferation (Franco P. and Stoppelli M.P., Signaling effects of Pep 2 via αv-Integrin, unpublished). If the reduced HT1080 cell invasion is due to the lower number of si-αv TIFs in the matrices or to a reduced αv-dependent pro-invasive ability remains to be determined.

To verify the presence of secreted motogen factors following RNA silencing in TIF fibroblasts, as previously observed for peptide-treated fibroblasts, CM from αv-silenced TIFs were employed as chemoattractants for HT1080-GFP in directional migration assays. Figure 6C shows an approximately 2.5-fold increase in HT1080-GFP chemoattraction by CM from TIFs carrying si-CTRL. In contrast, CM from αv-silenced TIFs lost the ability to chemoattract HT1080-GFP cells, showing a phenotype similar to that observed in Figure 3D. Collagen reorganisation by αv-silenced TIFs was then tested by a collagen I matrix contraction assay, 24 h after transfection with si-CTRL or αv integrin siRNA. TIFs were processed as in Figure 5A, and time-dependent decrease of matrix area, was monitored. Figure 6D shows that αv-silenced TIFs generated matrices with 30% and 10% larger areas 2 and 3 days after siRNA transfection, respectively, as compared to fibroblasts carrying si-CTRL. Overall, these findings show that either treatment with both decapeptides or αv-silencing reduce the secretion of motogen factors and impair the matrix contractile ability of TIF fibroblasts.

Then, the efficiency of αv integrin RNA silencing and the concomitant levels of α-SMA were evaluated by western blot. As shown in Figure 6E, the 50% reduction in αv protein levels after 48 h of silencing, and the 70% after 72 h was accompanied by a reduction in the expression levels of α-SMA protein by 60% after 48 or 72 h.

In addition, α-SMA levels were assayed in αv-lacking TIF fibroblasts by immunofluorescence assays and quantified by ImageJ software (Figure 6F). In accordance with the results shown in Figure 6E, αv-silencing causes a 50% decrease in α-SMA protein levels in peptide-exposed TIF fibroblasts, confirming that treatment with peptides and αv-silencing produce similar intracellular effects. Overall, these data further support the peptide-αv interaction and highlight a relevant role for αv integrin as a mediator of CAF phenotype down-modulation.

### 2.9. Exposure to Pep 2 of Primary CAFs Prevents Their Pro-Invasive Activity in a 3D-Assay

Many studies have confirmed the active role of CAFs in breast TME and their effects on the onset, growth and spread of neoplastic cells [4]. Considering that the novel decapeptides can partially revert the CAF-like phenotype of TIFs by interfering with their pro-invasive ability and CAF markers expression, an effort was made to extend these results to primary breast CAFs. Intra-tumoral fibroblasts were previously isolated from biopsies of two breast adenocarcinoma samples, from patients H and M (H-CAFs and M-CAFs, respectively) by De Vincenzo et al. [6]. Firstly, these primary CAFs could contract collagen matrices, similarly to that observed with TIFs (Franco P. and Stoppelli M.P., Signaling effects of Pep 2 via αv-Integrin, unpublished). This finding opened the possibility to test CAFs susceptibility to Pep 2 in a 3D-organotypic assay with primary breast CAF and the highly aggressive, poorly differentiated MDA-MB-231 breast adenocarcinoma cells, to recapitulate mammary tumor-CAF interactions.

**Figure 7:**
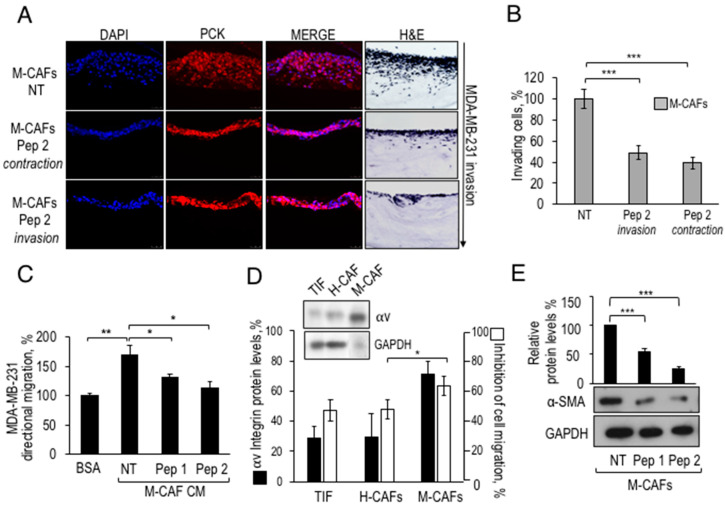
Inhibition of primary breast CAFs pro-invasive activity by Pep 2. (**A**) Organotypic assay with 5 × 10^4^ M-CAFs, pre-exposed for 1 h at 37 °C to 100 nM Pep 2 or diluents in DMEM-10% FBS, mixed with neutralized collagen I. The assay includes either diluents (NT), or 100 nM Pep 2 either during contraction (Pep 2 contraction) or invasion (Pep 2 invasion). Treatment during matrix contraction was for 3 days/week, for 2 weeks and then the peptide was excluded from subsequent invasion. Then, 1 × 10^5^ MDA-MB-231 cells were seeded on top of matrices for three days. The invasion assay was performed in the presence (Pep 2 invasion) or in the absence (Pep 2 contraction) of 100 nM Pep 2, for 6 days. After paraffin embedding, matrices were DAPI stained and analyzed by immunofluorescence with Anti-Pan-Cytokeratin antibody. (**B**) The number of invading cells was quantified by Image software, the number of MDA-MB-231 invading cells in untreated matrices was considered as 100%. (**C**) 5 × 10^4^ M-CAFs were seeded in DMEM-10% FBS and CM were prepared as described in the legend of Figure 3D and employed as chemoattractants for MDA-MB-231 cells in Boyden chamber migration assays. In each sample, 2 × 10^4^ MDA-MB-231 cells were tested either toward BSA-0.1% (basal migration) or toward M-CAFs CM. (**D**) Correlation between αv integrin protein levels in TIF, H-CAFs and M-CAFs and their sensitivity to Pep 2-dependent inhibition of cell migration. Inset: Western Blot analysis of αv protein levels in total cellular lysates of TIF, H-CAFs and M-CAFs. Black columns in the histogram show the relative densitometric analysis. White bars represent the % of Pep 2-dependent inhibition of TIFs or H-CAFs or M-CAFs migration. 5 × 10^4^ fibroblasts were treated for 1 h with 100 nM Pep 2 or with diluents and tested toward 3% FBS for 3 h at 37 °C. The results are reported as % of cell migration inhibition, relative to the FBS-dependent migration of untreated samples. (**E**) α-SMA protein levels analyzed by Western Blot in CAFs exposed to Pep 1 or Pep 2. 5 × 10^4^ M-CAFs were seeded in 6 well plates in DMEM-10% FBS, grown for 24 h, serum-starved for 6 h, and then treated with 100 nM Pep 1 or Pep 2 or diluents in DMEM-10% FBS. Peptides were re-added to the medium after 24 h and total cellular lysates were prepared after 48 h. Densitometric analysis of Western Blot is shown on top. * *p* < 0.05; ** *p* < 0.005; *** *p* < 0.001, Student’s *t*-test. The whole blot images can be found in Figure S9.

The specific binding of FITC-Pep 2 to intact MDA-MB-231 cells encouraged further functional testing (Figure S4A). Although proliferation of MDA-MB-231 exposed to Pep 2 is not affected, cell migration and matrigel invasion are definitely prevented by Pep 2 (Figure S4B–D). To investigate the effects of Pep 2 in the mammary TME context, M-CAFs were pre-treated with Pep 2 (contraction) or diluents (NT), before to be mixed with neutralized collagen type I solution. The contraction phase lasted 2 weeks, and fresh Pep 2 was included every 3 days. Then, Pep 2 was removed, and MDA-MB-231 tumor cells were seeded for starting the invasion phase, in presence (invasion) or in absence of Pep 2 (contraction). After 6 days, evaluation of breast adenocarcinoma invasion was carried out, as described for the assay shown in Figure 3B,C, and matrix sections were analyzed by immunofluorescence with monoclonal anti-cytokeratin pan mixture (Pck), to distinguish epithelial MDA-MB-231 cells from M-CAFs, not expressing this epithelial marker (Figure 7A). As shown in Figure 7B the number of invading MDA-MB-231 cells decreased by 50% after 6 days of invasion in the presence of Pep 2, compared to control MDA-MB-231 (Figure 7B). If CAF-matrices are exposed to Pep 2 only during the contraction phase, an about 50% decrease of mammary cell invasion resulted, suggesting a relevant contribution of matrix fibroblasts to the inhibition of tumor invasion. The finding that M-CAFs express about 80% of the αv integrin expressed by MDA-MB-231 well agrees with the robust inhibitory effect of Pep2 included in the contraction phase.

Next, we investigated whether the peptide-dependent impairment in mammary tumor invasion may be caused by a decreased secretion of motogen factors by Pep 2-treated CAFs, as observed for peptide-exposed and αv-lacking TIFs. Therefore, the CM of M-CAFs fibroblasts treated with either Pep 1 or Pep 2 were collected and employed as chemoattractants for MDA-MB-231 in directional migration assays. Figure 7C shows that CM from peptide-treated fibroblasts lost the ability to chemoattract breast adenocarcinoma cells. To compare αv integrin expression levels and sensitivity to the inhibition by Pep 2, a directional migration assay with mammary H-CAFs and M-CAFs was set up. We found that M-CAFs were the most sensitive to Pep 2 inhibition (Figure 7D). The inset to Figure 7D shows a comparison among αv integrin protein levels of TIFs, H-CAFs and M-CAFs, highlighting that M-CAFs lysates also contain the highest level of αv integrin. Finally, exposure of M-CAFs to Pep 1 or Pep 2 induces a 50% and 70% decrease of α-SMA protein levels, respectively (Figure 7E). These findings suggest that breast CAFs expressing αv integrin may be targeted by the novel decapeptides, and that this interferes with secretion of chemotactic factors, α-SMA expression, and ability to elicit invasion of breast carcinoma cells.

## 3. Discussion

In the present study, we investigated the anti-migratory and anti-invasive effects of two urokinase-derived, novel decapeptides, co-targeting tumor cells and stromal fibroblasts.

Firstly, the cyclic Pep 2 could significantly reduce the number and the size of HT1080 fibrosarcoma cells metastases to the lungs of nude mice. Second, both peptides proved to be potent inhibitors of myofibroblasts in 3D-organotypic co-cultures, as peptide-exposed TIF fibroblasts or CAF from breast carcinoma patients lose their ability to stimulate matrix invasion of HT1080-GFP fibrosarcoma or MDA-MB-231 mammary tumor cells, respectively. Third, following exposure to either peptides, CAFs exhibit decreased α-SMA levels, and to mobilize tumor cells.

Also, specific binding assays with cells expressing reduced or enhanced αv levels identified αv-integrin as the Pep 2 molecular target. Finally, TIFs, either exposed to Pep 2, or silenced for αv integrin, exhibit a reduced ability to chemoattract cancer cells and to contract collagen matrices, together with reduced α-smooth muscle actin (α-SMA) levels. In conclusion, the novel peptides, neither affect the extent of HT1080 and TIFs proliferation, nor the level of apoptosis. Also, they do not interfere with HT1080 or TIFs cell adhesion (Franco P. and Stoppelli M.P., Signaling effects of Pep 2 via αv-Integrin, unpublished). In contrast, these compounds strongly inhibit migration and invasion of HT1080 and MDA MB-231 cells, as well as migration, matrix contraction and α-SMA expression of TIFs and primary CAFs. These data uncover the ability of two novel peptides to counteract tumor invasion through binding to the αv integrin subunit and down-modulation of the CAF phenotype.

As heterodimeric primary receptors in cell-matrix adhesion, integrins recognize binding motifs in ECM proteins, but they can also promote stemness and survival in a ligand-independent manner [10]. Among them, the heterodimeric αv integrins, namely αvβ3, αvβ5, αvβ6, and αvβ8 are overexpressed in primary bladder, colorectal, breast, lung, renal and melanoma tumors, and expressed at higher levels in the corresponding brain metastases [11]. In particular, the integrin subunit αv gene *ITGAV* is overexpressed and associated with progression and spread of colorectal cancer [19]. Overexpression of *ITGAV* is associated with poor relapse free survival of breast cancer patients. Silencing of *ITGAV* inhibited cell proliferation, invasion, and self-renewal of breast cancer cell lines by altering expression of *BCL2* and *PXN* [20].

The decapeptides presented here bind to αv integrin subunit and inhibit migration, invasion and in vivo dissemination of fibrosarcoma and mammary adenocarcinoma cells. Target specificity is shown by the reduced binding of FITC-Pep 2 following αv-interference, or exposure to anti-αv blocking antibodies as well as increased peptide binding following αv overexpression. Furthermore, we show that silencing of αv integrin expression in TIF fibroblasts leads to an impairment of their matrix contraction ability, to a decrease in the contractile α-SMA protein levels and to the inhibition of fibroblast ability to stimulate invasion of fibrosarcoma and breast adenocarcinoma cells. These findings indicate the occurrence of an αv-dependent, partial loss of CAF-like phenotype, as shown by the α-SMA decreased and CAV-1 increased levels [21]. The strict similarities of the functional effects of αv reduced expression, and those observed in peptide-exposed TIF further support the central role of αv integrin in our system.

In human breast tumors, highly metastatic and poor clinical outcomes are associated with ECM stiffening, also depending on the activation of mechanotransduction pathways through integrin-dependent signaling [22]. Indeed, the myofibroblast-like properties of CAFs are relevant to the generation of a stiff ECM within the TME that supports invasive tumor growth [23]. Interestingly, the ability of fibroblasts to contract connective tissue matrices generates tractional forces, and a rigid ECM, that is sensed by tumor cells migrating preferentially toward stiffer surfaces. Not only chemotactic gradients, but also increased local ECM stiffening may cause increased migration toward the areas of higher ECM rigidity via mechanosensing, a mechanism by which cells convert mechanical stimuli into signal transduction activity [24]. Here, we show that peptide-exposed TIF fibroblasts are impaired in their matrix contraction ability, producing softer matrices than the untreated counterparts (Figure 5A,B and Figure 6D). This finding indicates that Pep 1 and Pep 2 interfere with CAF ability to establish a pro-metastatic environment, also considering that matrix stiffness and crosslinking is associated to enhanced integrin signalling and tumor progression [25]. Therefore, the ability of Pep 1 and Pep 2 to reduce matrix stiffness of TIF fibroblasts may account, at least in part, for their impaired pro-invasive capacity (Figure 4). In general, the two novel decapeptides induce a partial reversion of the CAF-like phenotype, including the ability to produce stiff matrices.

In general, the activity of peptides has received increasing attention: peptides display a greater efficacy, selectivity and specificity than small-molecule drugs, with few off-target effects. Moreover, peptides are applicable as lead compounds for pharmacophores or to the design of drug-like molecules with incorporated secondary structural elements. Pep 1 and Pep 2 decapeptides share some similarities with an 8-mer capped peptide corresponding to residues 136–143 of human uPA and denoted Å6 (Table 1). This peptide is endowed with a clear-cut biological activity, as it inhibits angiogenesis and metastases of rat breast cancer cells [26]. The clinical studies indicated that in phase I trials, in gynaecologic malignancies, Å6 was well tolerated, without any immunogenic response. Importantly, a randomized, double-blind, phase II study pointed to a statistically significant delay in time to clinical progression [27,28]. Although the authors report a specific interaction of Å6 with CD44, we have previously shown that Å6 competes with CPp (corresponding to residues 135–158 of human uPA) for binding to αv integrin, suggesting that all peptides, derived from the N-terminal region of uPA CP, share the same target integrin [15]. Moreover, Baggio et al. [29] have found that Å6 does not interact significantly with recombinant hCD44(21–178). Pep 1 was designed on the basis of the 3D structure of our previously published uPA-derived peptides [15] and Pep 2 may be considered as a conformationally constrained analog of Pep 1. Elongation of Å6 to Pep 1 and its subsequent cyclization to Pep 2, aimed at stabilizing the active conformation of anti-migratory Å6-like peptides, led to a 30-fold decrease in the IC_50_ in HT1080 cell migration assays (Stoppelli M.P.; Carotenuto A.; et al.; Patent n.10201800010511). Peptide cyclization results in a further 3-fold decrease in the IC_50_, as shown in Figure S1A. In fact, the well-defined 3D structure of Pep 1 consists in a β-turn with a tip on residues Pro141-Glu142 flanked by two strain regions connected by a salt bridge involving the side chains of Glu138 and Lys145 (Figure 1). The peptides presented here have a high affinity for the target: in migration assays with HT1080 fibrosarcoma, with breast adenocarcinoma MDA-MB-231 and TIF fibroblasts, the IC_50_ of uPAcyclin ranges from 5 to 100 pM, all definitely lower than the reported µM concentrations of the small cyclic pentapeptide cilengitide [30].

In this study, we have not determined the identity of the integrin β monomer/s associating to αv subunit in the cells examined; however, in our previous studies we have shown that uPA-derived peptides interact with αvβ5 and not with αvβ3 [17]. In TME, integrins modulate CAFs ability to generate and to respond to paracrine signals generated by the epithelial components. Here, we show that αv integrin is expressed by primary breast CAFs and that its expression level correlates with the extent of inhibitory response to the uPAcyclin (Figure 7D). Furthermore, organotypic assays show that cancer cells fail to invade collagen matrices if collagen-embedded CAFs were pre-exposed to either peptides (Figure 3B and Figure 7A). These findings encourage targeting of αv-expressing CAFs with the novel decapeptides, as a therapeutic anti-cancer strategy.

Among the anti-integrin drugs, the cyclic pentapeptide cilengitide, binding to the RGD region and selective for αvβ3 and αvβ5 integrins, has been tested in clinical trials. Although this drug was well tolerated ad could be safely administered to cancer patients, the results have been discouraging [31]. Among other anti-cancer drugs targeting integrins like αvβ1, αvβ3, αvβ5, αvβ6, αvβ8, it has been proposed the therapeutic use of anti-αv antibody abituzumab, in colorectal and prostate cancer [32], and intetumumab [33]. For α5β1 integrin, the anti-α5 volociximab is available. Clinical studies have shown that they are inefficient in oncological patients, perhaps because of functional redundancy, promiscuity and compensation that extend the effects of the inhibitors in an unwanted manner. A few studies encourage to be cautious, because the continuous administration of low dosage RGD peptides, may stimulate tumor growth and angiogenesis, promoting endothelial induced by VEGF [34]. Despite the failure of anti-cancer treatments based on integrins, current knowledge does not allow to exclude their relevance as targets [12]. An interesting possibility would be to use anti-αv integrins in solid tumors expressing high levels of αv integrin and abundant stromal αv-enriched CAFs. In general, targeting the TME for more efficient anti-cancer therapies is a hot topic, especially for lethal malignancies where the stromal involvement is well recognized, like in the pathogenesis of ovarian cancer [35]. Evidence that treatments targeting both the tumor epithelia and the surrounding CAFs can extend the efficacy of conventional chemotherapies is provided by the retinoic acid receptor β and androgen receptor antagonists identified for concurrent therapy with cisplatin [36]. FAP-α, is a major target in TME with the oral proteolytic inhibitor talabostat [37]. A combination therapy of doxorubicin with pirfenidone, an antifibrotic agent and a TGFβ antagonist has great potential for the therapy of triple negative breast cancer targeting tumor-stroma interactions [38]. Our previous results indicated the relevance of Insulin-like growth factor-1 (IGF-1) and Insulin-like growth factor-1 (IGF-2) in the early breast epithelial-fibroblasts crosstalk, suggesting the therapeutic efficacy of OSI-906, a tyrosine kinase inhibitor of IGF-1R [6]. In general, understanding the complex interactions engaged by the tumor cells with the surrounding microenvironment may results in more effective co-targeting therapeutic strategies, that may ultimately improve patient outcomes.

## 4. Materials and Methods

### 4.1. Cell lines and Culture Conditions

The HEK-293 human embryonic kidney cell line was purchased at ICLC Interlab Cell Line Collection (Genoa, Italy). HEK-293-αv, clone 38 cells were obtained after stable trasfection of HEK-293 with pcDNA3-αv as described previously [14]. The HT1080 human fibrosarcoma cell line was also purchased from the ICLC Interlab Cell Line Collection. HT1080-GFP cells were obtained after stable trasfection of HT1080 cells with pEGFP-N1 vector (Clontech-Takara Bio, Mountain View, CA, USA), encoding green fluorescence protein (GFP). MDA MB-231 cell line derived from a human breast adenocarcinoma, was a gift of M.V. Carriero, National Tumor Institute IRCCS-Fondazione Pascale, (Naples, Italy). P19 cell line derives from a murine embryonic teratocarcinoma and was provided by A. Cimmino, IGB-CNR (Naples, Italy). Telomerase-immortalised primary dermal fibroblasts or TIF, derived from biopsies of human forearm, were a gift of Prof. P. Timpson, Garvan Institute of Medical research (Sydney, Australia) [39]. CAFs were isolated from two breast adenocarcinoma patients (M and H) and described in a previous study [6]. All cell lines and primary fibroblasts were cultured in DMEM or RPMI supplemented with 10% fetal bovine serum (FBS) and penicillin (100 U/mL)/streptomycin (100 μg/mL), at 37 °C, under 5% CO_2_. All cell culture reagents were purchased from Gibco (Gaithersburg, MD, USA).

### 4.2. Peptide Synthesis

Peptides were synthesized by using the ultrasound-assisted solid-phase peptide synthesis (US-SPPS) Fmoc-based strategy [40]. In particular, the linear peptide elongation was performed on the Rink amide resin as solid support, by featuring cycles of Fmoc-deprotection (20% piperidine in DMF solution (0.5 + 1 min) by ultrasonic irradiation) and coupling reaction (Nα-Fmoc-amino acid (2 equiv), COMU/Oxyma (2 equiv each), and DIEA (4 equiv) by ultrasonic irradiation for 5 min) steps. After each reaction, the resin was washed with N,N-Dimethylformamide (DMF) (3 × 2 mL) and Dichloromethane (DCM) (3 × 2 mL), and the process was monitored by specific colorimetric assays [40]. Pep 2, carrying the allyl (All)-derived protecting groups on Glu and Lys side chains, was subjected to cyclization on solid-phase to accomplish the lactam bridge, as elsewhere described [41,42]. Briefly, all and allyloxycarbonyl (Alloc) groups were simultaneously hydrolyzed by a treatment with a solution of Pd(PPh_3_)_4_ (0.15 equiv) and N,N′-dimethylbarbituric acid (NDMBA) (7 equiv) in DCM/DMF (3:2 v/v), for 2 h at room temperature. The resins were filtered and washed, and the allyl-deprotection procedure was repeated once again. After complete removal of the allyl groups, the coupling was performed by using PyAOP (2 equiv) and HOAt (2 equiv), in the presence of DIEA (4 equiv), for 2 h at room temperature on an automated shaker. The peptide-resin was washed with DCM (5 × 2 mL) and dried. Finally, the Pep 1 and Pep 2 N-terminal ends were acetylated, whereas that of FITC-Pep 2 was conjugated to fluorescein, as reported by Jullian et al. [43].

The resin-bound peptides were treated with a cocktail cleavage (TFA/TIS/H_2_O 95:2.5:2.5 (v/v/v)), at rt for 3 h, to yield crude peptides and their purification was performed by HPLC on a preparative RP C18 column (Kinetex, 5 μm, 150 × 21.2 mm, 100 Å, Phenomenex, Torrance, CA, USA) using specific linear gradients of MeOH (0.1% TFA) in water (0.1% TFA) with a flow rate of 10 mL/min (from 10 to 90% over 30 min) and UV detection at 220 nm. Final products were obtained by freeze-drying the appropriate fractions after removal of MeOH under reduced pressure by rotary evaporation. The purity of compounds was ascertained by analytical HPLC analyses, which were performed by a Prominance system (Shimadzu, Kyoto, Japan) equipped with a Phenomenex Kinetex column (C18, 150 mm × 4.6 mm, 5 μm, 100 Å), and a flow rate of 1.0 mL/min, with detection at 220 nm wavelength by a diode array UV-Vis detector, and by using a gradient elution of MeOH (0.1% TFA) in water (0.1% TFA), over 20 min. All peptide compounds examined for biological activity were purified to >98%, and the correct molecular ions were confirmed by HRMS measurements (LTQ Orbitrap, ThermoFisher Scientific, Waltham, MA, USA) in positive ESI mode.

### 4.3. Conformational Analysis

NMR Spectroscopy. Samples were prepared by dissolving peptides in 0.54 mL of H_2_O and 0.06 mL of D_2_O (pH 5.5), to obtain a 1 mM final concentration. NMR spectra were recorded on an INOVA 700 MHz spectrometer (Varian, Palo Alto, CA, USA) equipped with a z-gradient 5 mm triple-resonance probe head at 25 °C. One-dimensional (1D) NMR spectra were recorded in the Fourier mode with quadrature detection. The water signal was suppressed by gradient echo [44]; 2D DQF-COSY [45,46], TOCSY, [47] and NOESY [48] spectra were recorded in the phase-sensitive mode using the method from States et al. [49]. Data block sizes were 2048 addresses in t2 and 512 equidistant t1 values. Before Fourier transformation, the time domain data matrices were multiplied by shifted sin^2^ functions in both dimensions. A mixing time of 80 ms was used for the TOCSY experiments. NOESY experiments were run with a 100 ms mixing time. The qualitative and quantitative analyses of DQF-COSY, TOCSY, and NOESY spectra, were obtained using the interactive program package XEASY [50]. Almost complete ^1^H-NMR chemical shift assignments were effectively achieved for Pep 1 and for the main conformer of Pep 2 according to the Wüthrich [51] procedure via the usual systematic application of DQF-COSY, TOCSY, and NOESY experiments with the support of the XEASY software package (Tables S1 and S2). The NOE-based distance restraints were obtained from NOESY spectra of Pep 1 (Table S3). The NOESY cross peaks were integrated with the XEASY program and were converted into upper distance bounds using the CALIBA program incorporated into the program package CYANA [52]. Only NOE derived constraints were considered in the annealing procedures. An ensemble of 100 structures was generated with the simulated annealing of the program CYANA. Then, 10 structures were chosen, whose interproton distances best fitted NOE derived distances, and refined through successive steps of restrained and unrestrained energy minimization calculations using the Discover algorithm (Accelrys, San Diego, CA, USA) and the consistent valence force field [53]. The minimization lowered the total energy of the structures; no residue was found in the disallowed region of the Ramachandran plot. The final structures were analyzed using the InsightII program (Accelrys). Molecular graphics images were realized using the UCSF Chimera package [54].

### 4.4. Binding Assay

HEK-293, HEK-293-αv, TIF, HT1080 and MDA MB-231 cells were harvested and acid treated, as described in Stoppelli et al. [55]. Then, 2 × 10^6^ cells/sample were incubated in suspension with 50 nM FITC-Pep 2 in 100 µL, for 2 h at 4 °C. When indicated, cells were pre-incubated for 30 min at 4 °C with unlabeled Pep 2 or scrambled Pep 2 at the indicated concentrations, or with the following antibodies: VNR147 monoclonal anti-αv integrin and polyclonal anti-α3 antibodies (Chemicon Int. Inc., Temecula, CA, USA), polyclonal anti-αv (N-9, Santa Cruz biotechnology, Dallas, TX, USA), polyclonal anti-actin (A2066, Sigma-Aldrich, St Louis, MO, USA), monoclonal anti-GAPDH (Ab9484, Abcam, Cambridge, UK), or purified vitronectin (VN, Corning, Glendale, AZ, USA). FITC-Pep 2 peptide preparation retained 80% biological activity. At the end of incubation, cells were washed three times with PBS-0.1% BSA and cell surface-associated FITC-Pep 2 was determined using a fluorimeter (VICTORTM X3-PerkinElmer, Waltham, MA, USA).

### 4.5. Protein Extraction and Western Blot Analysis

Total protein extracts were prepared from whole cells as described [6], resolved by SDS-PAGE, blotted onto PVDF membranes (Millipore, Burlington, MA, USA) and probed with the following primary antibodies:

- polyclonal anti-α-SMA (Abcam; Ab5694)
- polyclonal anti-Caveolin-1 (Santa Cruz Biotechnology; sc-894)
- monclonal ant-GAPDH (Abcam; Ab9484)
- polyclonal anti-αv (Millipore; Ab1930)

As secondary antibodies, goat anti-rabbit IgG-HRP (Sigma; A-6154) or goat anti-mouse IgG-HRP (Santa Cruz Biotechnology; sc-2005) were used. The peroxidase activity was measured with ImmobilionTM Western, Chemiluminescent HPR Substrate (WBKLS0500, Millipore). Films were imaged using the CanoScan 4400F (Canon, Ohta-ku, Tokyo, Japan) at 300 dpi, with the Adobe Photoshop Creative Suite 2 or CS2 and bands quantified with the ImageJ 1.52a software (NIH, Bethesda, MD, USA).

### 4.6. Apoptosis Assay

Cell apoptosis was measured using Promega Caspase 3/7 Glo reagent (Promega, Milan, Italy). TIF fibroblasts or HT1080-GFP were seeded in 96 wells white plates for 24 h in DMEM-10% FBS and serum-starved for 6 h. Then, they were treated with DMEM-10% FBS or DMEM, in the presence or in the absence of 100 nM Pep 2. At the indicated time points, cells were lysed with 100 μL of Caspase-Glo 3/7 buffer, containing luminogenic caspase substrate (tetrapeptide sequence DEVD) and, after 15 min, the luminescent signal, that is proportional to the amount of caspase activity, was measured at a luminometer (GloMax^®^96, Promega), according to the manufacturer′s instructions.

### 4.7. RNA Silencing

Silencing of αv integrin was accomplished by the esiRNAs mixture (#EHU002301, Sigma-Aldrich) targeting human ITGAV (esiRNA1, NCBI accession no. NM-002210, https://www.ncbi.nlm.nih.gov/genbank/). Cells were transfected by the “fast-forward” protocol using HiPerfect (Qiagen, Hilden, Germany), according to the manufacturer’s instructions. Briefly, HT1080 cells (1.25 × 10^5^ cells/sample) or TIF fibroblasts (5 × 10^4^ cells/sample) were seeded in 6 well plates in DMEM-10% FBS, grown for 24 h and then transfected with a mixture containing 60 nM or 30 nM αv-targeting (for HT1080 or TIF, respectively) or control siRNAs in serum-free medium using HiPerfect. Protein expression was tested 48 or 72 h later by western blotting.

### 4.8. Tail Vein Assay of Lung Metastasis

To assess HT1080-derived lung metastases, 5–6-week-old female BALB/c (nu/nu) mice were employed (Charles River Laboratories, Milan, Italy). All animal experimental procedures were performed in agreement with national and international laws and guidelines on animal welfare and were approved by the Italian Ministry of Health-Animal Welfare Direction (Protocol No. DGSAF21940-A, approved on 16 November 2013. Animals were randomized into four experimental groups, including 5 mice each: Group 1, healthy animals; Group 2, injected with 1 × 10^6^ HT1080 cells at day 0 and at day 2; Group 3, mice treated at day 0 and day 2 with one dose of Pep 2 15 at mg/kg and after 1 h injected with 1 × 10^6^ HT1080 cells (pre-treated with 1 μM Pep 2 peptide at 37 °C for 1 h). Group 4, mice treated at day 0 and at day 2 with one dose of Pep 2 at 3.4 mg/kg and after 1 h injected with 1 × 10^6^ cells HT1080 previously treated with 1 µM Pep 2. After intravenous injection in the tail vein, all animals were subjected to treatment for 28 days with different drug regimens. Group 1 and group 2 animals were treated with vehicle (PBS 1×), whereas group 3 mice were treated daily with ten doses of Pep 2 at 15 mg/kg and, then, treated with one dose of Pep 2 at 15 mg/kg each six days. Group 4 mice were treated daily with 28 doses of Pep 2 at 3.4 mg/kg. Mice weight, breathing rate and overall health were daily monitored.

### 4.9. Ex Vivo Lung Analysis

After 28 days, mice were sacrificed by cervical dislocation and lungs were surgically removed. For each mouse, one lung was placed in liquid nitrogen and stored at −80 °C to be used for human DNA content quantitation (Section 4.10). Instead, the other lung was fixed in buffered 4% formaldehyde, paraffin embedded, 5 µm sectioned (RM2245, Leica Biosystems, Wetzlar, Germany) and stained with Hematoxylin and Eosin (H&E-staining) to observe intra-parenchymal and sub-pleural lung metastatic foci. Lung sections were viewed using Digital Image Hub 12.3.3.7055 software (Leica Microsystems) and images were captured at 4× magnification.

### 4.10. Quantification of Human DNA in Murine Lungs

Genomic DNA from the whole lung samples was extracted using RNase A, proteinase K and phenol-chloroform. 50 ng of DNA were used as template for Semi-Quantitative PCR reactions (T100 Thermal Cycler, Bio-Rad Laboratories, Milan, Italy). The DNA was incubated in a total reaction volume of 20 μL, containing 1× Reaction Buffer, Taq DNA polymerase (Cat #EME010001, EuroClone, Milan, Italy), 200 μM dNTP mixture and 5 mM MgCl_2_. Primers targeting human Alu sequence: (FW 5′-CACCTGTAATCCCAGCACTTT-3′/RW 5′-CCCAGGCTGGAGTGCAGT-3′) were employed to a final concentration of 0.5 μM. PCR products were separated by agarose electrophoresis, and the bands quantified through the ImageJ software. The number of human cells was calculated by comparing the PCR amplification signals with a standard curve, included in every run, generated by mixing from 1 to 1 × 10^7^ HT1080 cells with P19 embryonic teratocarcinoma cells, keeping 1 × 10^7^ as total cell number. The signal from the 1 × 10^7^ HT1080 sample was considered as 100% and DNA from healthy murine lung samples was included in every run as a control. Quantitation of three independent PCR amplification samples, separated on 1% agarose gels is accomplished by ImageJ software and Student *t*-test.

### 4.11. Immunofluorescence

Fibroblasts were fixed in 4% paraformaldehyde (PFA), permeabilized with 0.1% Triton X-100 and incubated with anti-α-SMA overnight at 4 °C. Then, cells were stained with secondary goat anti-rabbit IgG H&L AlexaFluor-488 (A-21204, Life Technologies, Carlsbad, CA, USA) as described in [56]. Parallel staining with irrelevant primary antibody or secondary antibody were used to control specificity. To stain nuclei, Vectashield mounting medium with DAPI (62248, ThermoFisher Scientific, Waltham, MA, USA) was used. Captured images were processed through ImageJ software and the mean fluorescence intensity (MFI) was measured. For immunofluorescence on matrix sections, 5 μm histological sections were first de-paraffinized and the antigen retrieval was performed by Tris/EDTA (10 mM Tris, 1 mM EDTA) 0,05% Tween 20 pH 9 for 2 h at 60 °C. Sections were incubated for 1 h in humidified room with blocking solution (1% Milk, 10% Goat Serum, 1% BSA, 1% NaN_3_, 0,1% Triton X-100, in PBS 1×), then, incubated at 4 °C over night with monoclonal pan-cytokeratin antibody (mouse monoclonal pan mixture clones C-11, PCK-26, CY-90, KS-1A3, M20, A53-B/A2, C25-62 (Sigma-Aldrich; C2562) diluted 1:400 in blocking solution. After three washes with PBS 1×, sections were incubated with monoclonal donkey anti-mouse IgG Alexafluor 594 (Abcam), 1 h at room temperature (1:400 in blocking solution). The nuclei were visualized by DAPI staining, incubated 10 min at room temperature and sections were mounted with hydrophilic Mowiol 488. Sample observations have been performed at the Leica DMI600B fluorescence microscope at various magnifications for the production of images subsequently analyzed with the software “Leica Application Suite Advanced Fluorescence Software” (Leica Microsystems).

### 4.12. Migration Assays

Boyden chambers assays were performed using 8 µm-pore-size PVDF-free filters coated with collagen type VI in directional migration assay, as described previously [6,15]. Briefly, HT1080, HT1080-GFP or TIFs were pre-treated for 1 h with Pep 1 or Pep 2 at the indicated concentrations, as described in the relative figure legends. Cellular migration was tested for 3 h at 37 °C toward DMEM-3% FBS, or serum-free TIFs CM. For invasion assays, filters were further coated with Matrigel 50 µg/mL (Sigma-Aldrich), and HT1080 cells invaded for 5 h at 37 °C towards DMEM-3% FBS. At the end of both assays, cells on the lower filter surface were fixed, stained with haematoxylin and counted under an inverted microscope at 20× magnification. For scratch wound healing assay, TIFs or HT1080 were grown until confluence in 12 multi-well plate containing culture inserts for live cell analysis (Ibidi GmbH, Martinsried, Germany) and pre-treated with Pep 1 or Pep 2 peptides or diluents for 1 h in serum-free medium for at 37 °C and then exposed to 1% FBS.

Wound healing assay was performed for 24 h, until scratch closing. Images were captured at the indicated time points at Leica DMI600B fluorescence microscope and analyzed with the Leica Application Suite Software (Leica Microsystems). The extent of wound closure is expressed as average decrease in wound distance in three points, considering as 100% the distance between the monolayer margins, at the time of wound formation.

### 4.13. Matrix Contraction Assay

TIFs (5 × 10^4^) were resuspended in DMEM-10% FBS, mixed with a neutralized collagen I solution, in the presence of the indicated peptides and the diameter of matrices was monitored. Matrix area with untreated TIFs at day 2 was considered as 100%. The 594-PSR picrosirius red staining of fibrillar collagen was performed according to Alcaraz et al. [57]. Sections were observed at 20× magnification and densitometric quantitation of fluorescence signal was performed with ImageJ software.

### 4.14. Organotypic Invasion Assay

Invasion of tumor cells through collagen I matrices including TIF or M-CAF cells was performed as described [6,58]. Briefly, fibroblasts were embedded in a neutralized collagen I solution, extracted from tendon of adolescent rat tails, and allowed to contract matrices in 35 mm plates in DMEM-10% FBS for 3 days (TIFs) or for a week (M-CAFs), in the presence or absence of Pep 1 or Pep 2, as indicated. Then, HT1080-GFP or MDA-MB-231 were grown on matrix top for 2 days in 24 well plates and matrices transferred to an air-liquid interface on a steel greed in 60 mm plates for the invasion phase. After 7 days, matrices were fixed in 4% PFA, paraffin embedded, sectioned and processed for H&E or DAPI (4′,6-diamidino-2-phenylindole) staining. MDA-MB-231 cells were stained with anti-pan-cytokeratin antibody (Sigma-Aldrich C2562) and with a secondary donkey anti-mouse antibody (Alexafluor 594). For quantitation of matrix invasion by HT1080-GFP or MDA-MB-231 cells, only images of fluorescence-emitting samples from a wavelength between 375 and 495 nm or 561 and 594 nm, respectively, were employed. Invading cells were counted in rectangular shapes selected on each matrix section image. The number of infiltrating cells into matrices was counted by ImageJ software and compared to untreated matrices and to the initial inoculus in the “seeding phase” (T0).

### 4.15. Statistical Analyses

Data are expressed as mean ± standard deviation of at least three separate experiments, indicated by error bars, performed in triplicate, unless otherwise specified. Differences between data sets were determined by the Student t test. Differences described as significant are indicated in the figures with *p* values ≤ 0.05 (*), ≤ 0.005 (**), or ≤ 0.001 (***).

## 5. Conclusions

Many current therapeutic approaches primarily target the growing solid tumors, largely ignoring the surrounding microenvironment. A great deal of findings has convincingly revealed the tumor promoting effects of CAFs, the most dominant cell type in TME. Here, we present two novel αv integrin binding decapeptides, inhibiting invasion of HT1080 and MDA MB-231 tumor cells as well as the pro-invasive activity of primary breast CAFs, reducing their α-SMA level and matrix contraction ability. These results may open new therapeutic perspectives on the use of these peptides as lead compounds for novel strategies to target breast CAFs.

## 6. Patents

Stoppelli M.P.; Carotenuto A.; et al.; “Novel peptides and peptidomimetics as potent targeted agents for prevention and treatment of tumor invasion and metastasis”. Italy, Patent n.10201800010511, 22 November 2018.

## Figures and Tables

**Table 1:** Sequences of relevant peptides.

Cmpd	Sequence *
CPp	Ac-K^135^KPSSPPEELKFQCGQKTLRPRFK^158^-NH_2_
[138E]uPA-(135–158)	Ac-K^135^KPESPPEELKFQCGQKTLRPRFK^158^-NH_2_
uPA-(135–143)	Ac-K^135^KPSSPPEE^143^-NH_2_
Å6	Ac-K^136^PSSPPEE^143^-NH_2_
Pep 1	Ac-K^136^PESPPEELK^145^-NH_2_
Pep 2 (uPAcyclin)	Ac-K^136^P[ESPPEELK^145^]-NH_2_
Scrambled Pep 2	Ac-PS[EELKPEPK]-NH_2_

* Square brackets indicate side-chain-to-side-chain cyclization. The numbering refers to the original sequence of the uPA protein.

## Supplementary Materials

The following are available online at https://www.mdpi.com/2072-6694/12/9/2404/s1, Figure S1: Inhibition of HT1080 wound healing closure by Pep 1 or Pep 2, Figure S2: Inhibition of TIF wound healing assay closure by Pep 1 or Pep 2, Figure S3: Unaffected proliferation and apoptosis of TIF fibroblasts and HT1080-GFP fibrosarcoma cells exposed to Pep 2, Figure S4: Interaction of Pep 2 with MDA-MB-231 breast adenocarcinoma cells, Figure S5: Uncropped image of the 1% agarose gel from Figure 2C, Figure S6: Whole blot image from Figure 4D, Figure S7: Whole blot image from Figure 5C, Figure S8: Whole blot image from Figure 6E, Figure S9: Whole blot image of Figure 7D,E, Table S1: NMR resonance assignments of Pep 1 in water solution at 10 °C, Table S2: NMR resonance assignments of Pep 2 in water solution at 10 °C, Table S3: NOE derived upper limit constraints of Pep 1.
