# Supplementary material for: Breast Tumor Cell Invasion and Pro-Invasive Activity of Cancer-Associated Fibroblasts Co-Targeted by Novel Urokinase-Derived Decapeptides
Source: Cancers (Basel). 2020 Aug 24;12(9):2404. doi: 10.3390/cancers12092404 (PMC7564779; doi:10.3390/cancers12092404)
Supplement: Supplementary file 1 [file cancers-12-02404-s001.pdf]

# Supplementary Material: Breast Tumor Cell Invasion and Pro-Invasive Activity of Cancer-Associated Fibroblasts Co-Targeted by Novel Urokinase-Derived Decapeptides

**Stefania Belli [1], Paola Franco, Francesca Iommelli, Anna De Vincenzo, Diego Brancaccio, Marialucia Telesca, Francesco Merlino [3], Ettore Novellino [3], Marie Ranson [4,5], Silvana Del Vecchio [6], Paolo Grieco, Alfonso Carotenuto and Maria Patrizia Stoppelli**

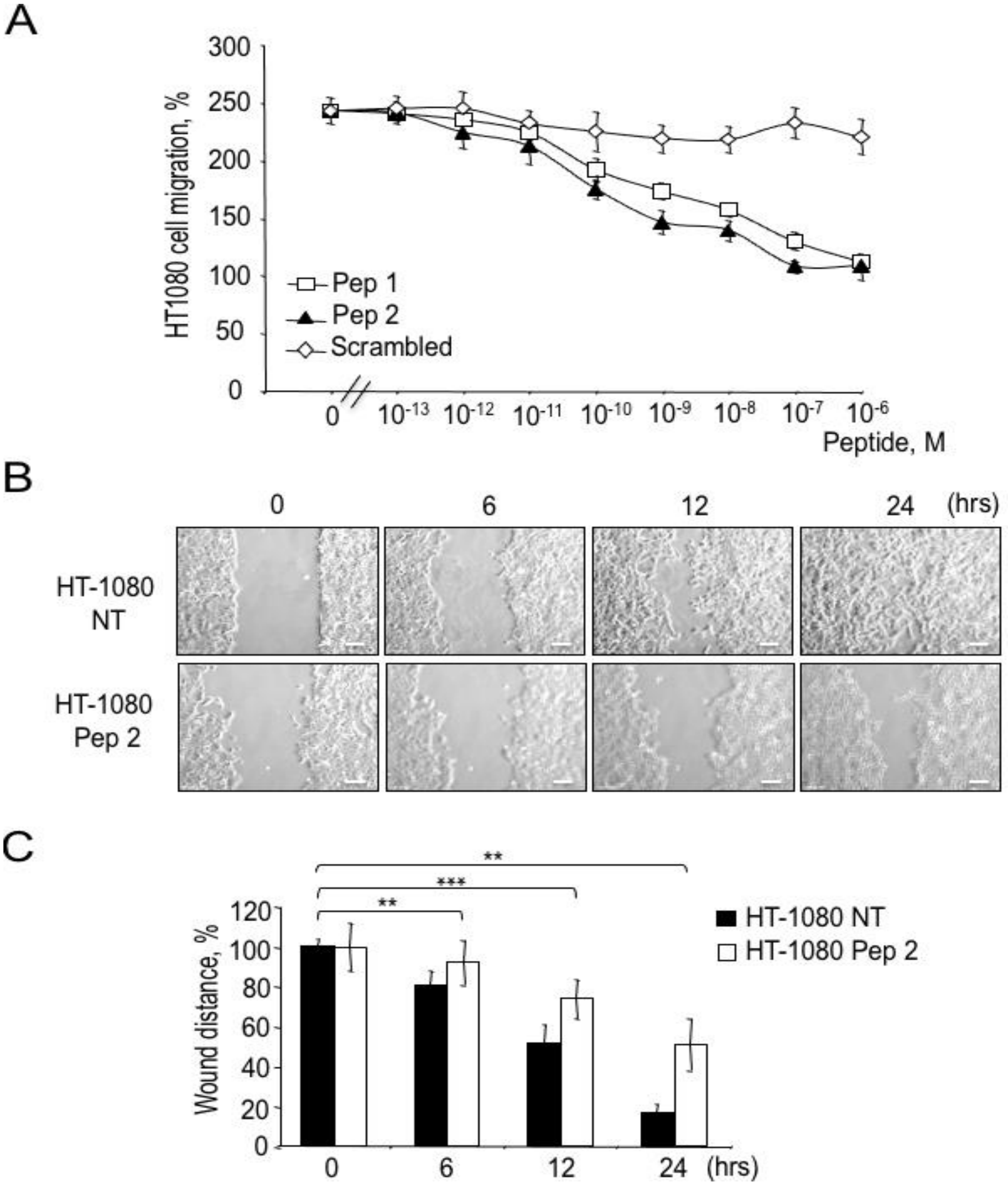


**Figure S1.** Inhibition of HT1080 wound healing closure by Pep 1 or Pep 2. (**A**) 2 × $10^4$ HT1080 cells/sample were pre-incubated with or without Pep 1, Pep 2 or scrambled Pep 2 peptides at the

indicated concentrations for 1 h at 37 °C and then allowed to migrate toward DMEM-1% FBS in Boyden chambers, as described in the legend to Figure 3D. (**B**) HT1080 cells were grown to confluence in 12-well plates with inserts causing a defined 500 µm cell-free scratch (ibidi, GmbH, Martinsried, Germany), pre-incubated with 100 nM Pep 2 or diluents in serum-free medium for 1 h at 37 °C and then exposed to 1% FBS, in the presence of Pep 2. Images were taken every 6 h for 18 h and representative images are shown. 20× Magnification, Scale bar, 50 µm. (**C**) The wound width at time 0 is taken as 100% and the average wound width at each time point is calculated as relative to that. Bars represent the average of three separate experiments undertaken in triplicate. Differences between data sets were determined by the Student's *t*-test, (** $p < 0.005$; *** $p < 0.001$, Student's *t*-test).

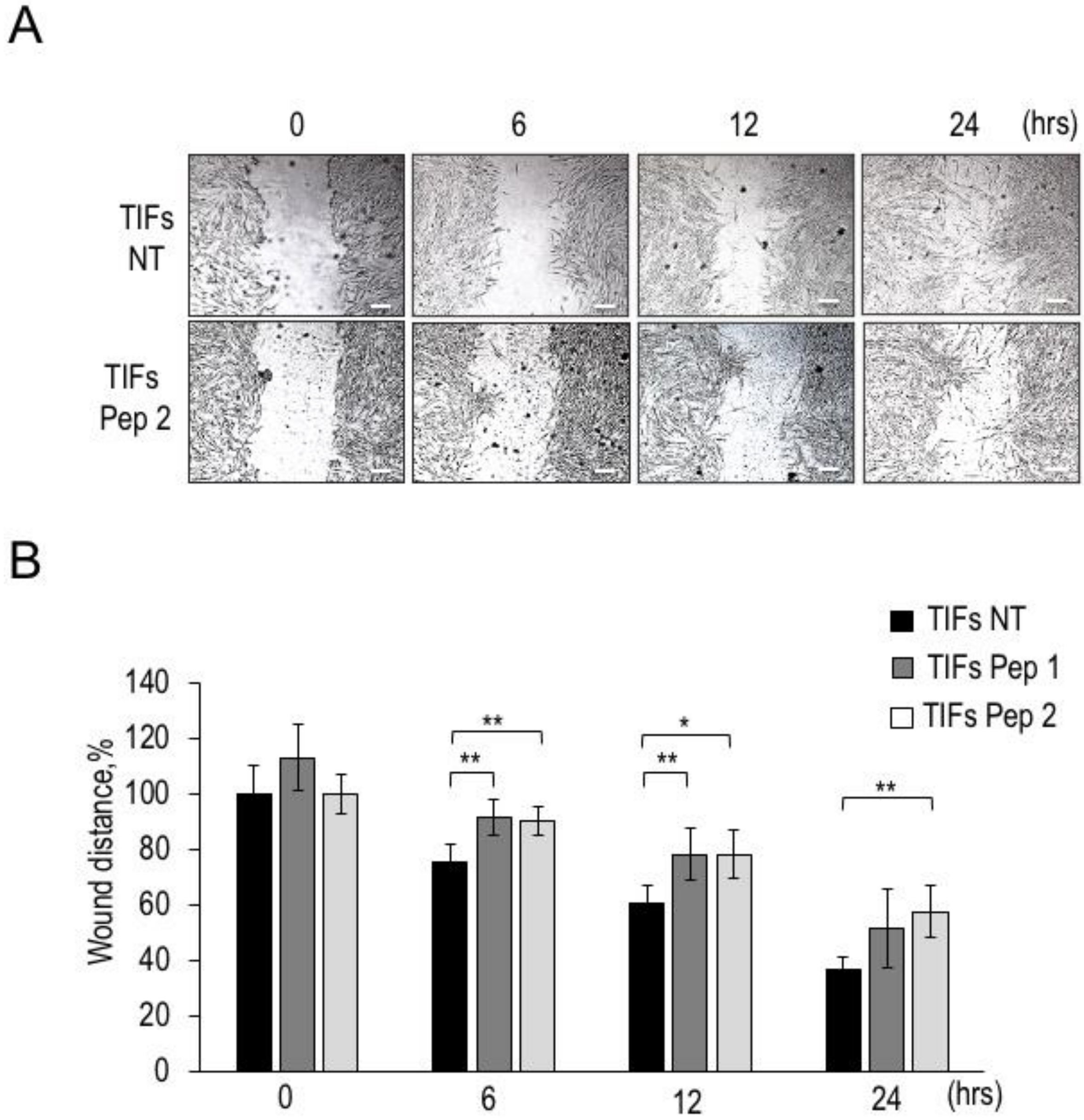


**Figure S2.** Inhibition of TIF wound healing assay closure by Pep 1 or Pep 2. (**A**) Wound healing assay of $1 \times 10^5$ TIF fibroblasts was conducted as described in the legend to figure S1B and monitored for the indicated time points. Representative images are shown, 20× Magnification, Scale bar, 50 µm. (**B**) The wound width was calculated as the average of three separate experiments undertaken in triplicate. (** $p < 0.005$; *** $p < 0.001$, Student's *t-test*).

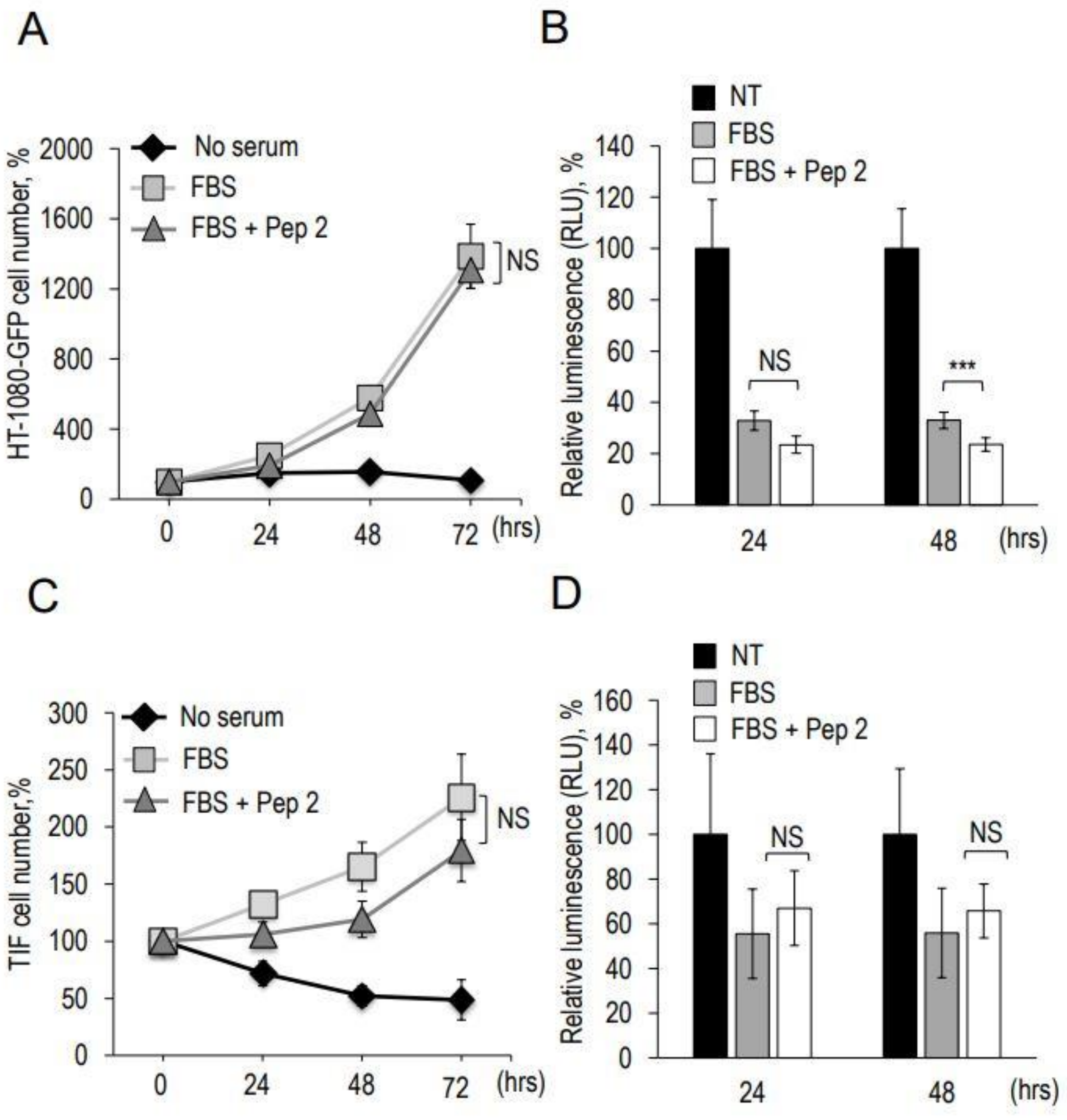


**Figure S3.** Unaffected proliferation and apoptosis of TIF fibroblasts and HT1080-GFP fibrosarcoma cells exposed to Pep 2. 2 × $10^4$ HT1080-GFP (**A**) or TIFs (**C**) were seeded in 12 well plates for 24 h in DMEM-10% FBS and serum-starved for 6 hrs. Then, cells were counted (0 h = 100%) and grown in DMEM (No serum) or DMEM-10% FBS (FBS), in the presence of Pep 2 (FBS + Pep 2), for 72 h (NS, non-significant). For the caspase 3/7 luminometric apoptosis assay, 2 × $10^3$ HT1080-GFP (**B**) or TIFs (**D**) were seeded in 96 well white plates for 24 h in DMEM-10% FBS, serum-starved for 6 h and grown in DMEM-10% FBS or DMEM, in the presence or in the absence of 100 nM Pep 2. Caspase 3/7 activity was measured by a luminometric assay after 24 and 48 h. Histograms represent the average of two separate experiments undertaken in triplicate (*** $p < 0.001$, Student's *t-test).*

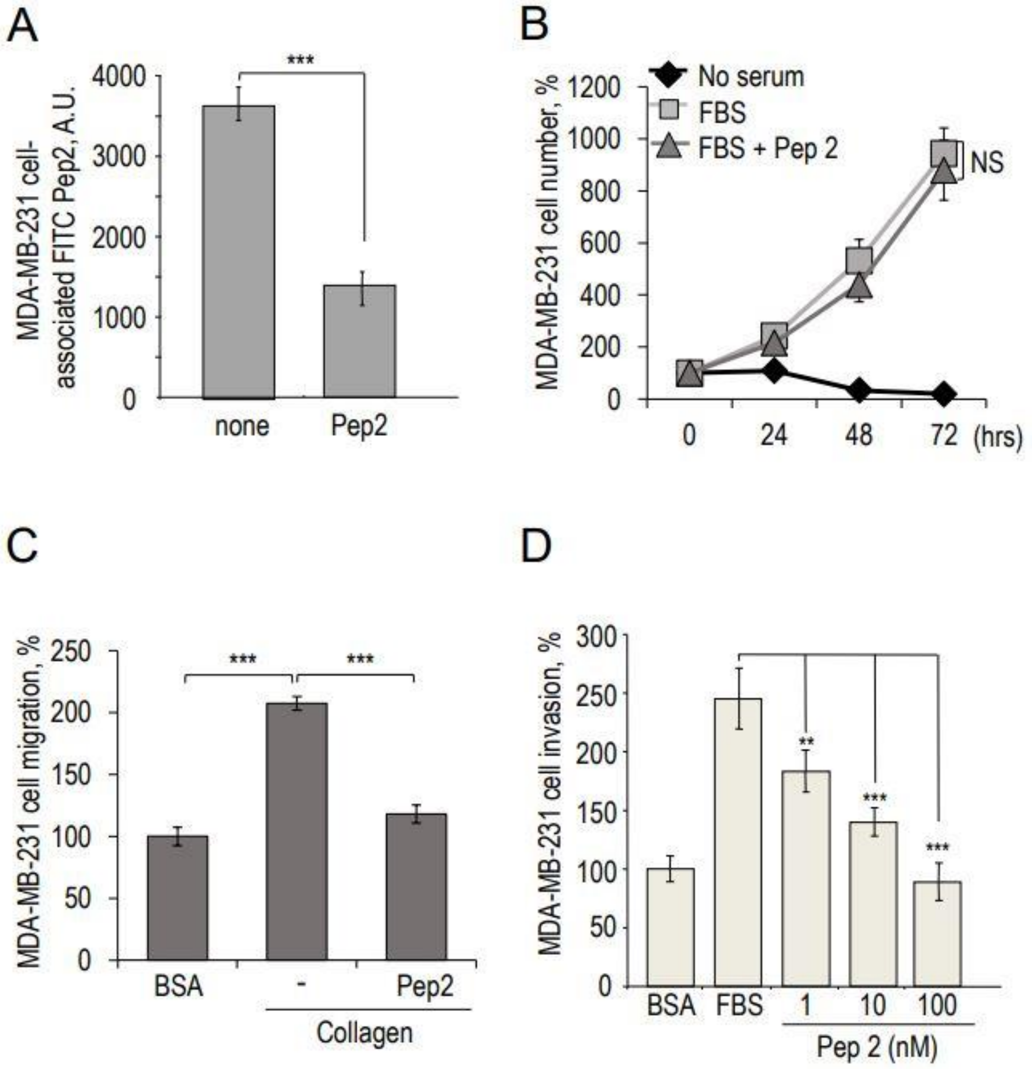


**Figure S4.** Interaction of Pep 2 with MDA-MB-231 breast adenocarcinoma cells. (**A**) 1.7 × $10^6$ cells/sample were assayed for FITC-Pep 2 binding, as described in Figure 4C. Data represent a mean of three independent experiments performed in duplicate (*** $p < 0.001$, Student's *t*-test). (**B**) 2.5 x $10^4$ MDA-MB-231 cells were seeded in 12 well plates for 24 h in RPMI-10% FBS and serum-starved for 24 h. Then, cells were counted (0 h = 100%) and incubated in RPMI (No serum) or RPMI-10% FBS (FBS) or in RPMI-10% FBS, in the presence of 100 nM Pep 2 (FBS + Pep 2), for 72 hrs. Cell counts represent the mean of three independent experiments performed in triplicate. (**C**) 2 × $10^4$ MDA-MB-231 were pre-treated for 1 h at 37 °C with 100 nM Pep 2 peptide or diluents and allowed to migrate toward 20 μg/mL collagen type VI. Data represent a mean of two independent experiments performed in triplicate (*** $p < 0.001$, Student's *t*-test). (**D**) 2 × $10^4$ MDA MB-231 cells/sample were pre-incubated with Pep 2 at the indicated concentrations and assayed for FBS-dependent invasion as described in Figure 4A (** $p < 0.005$; *** $p < 0.001$, Student *t*-test).

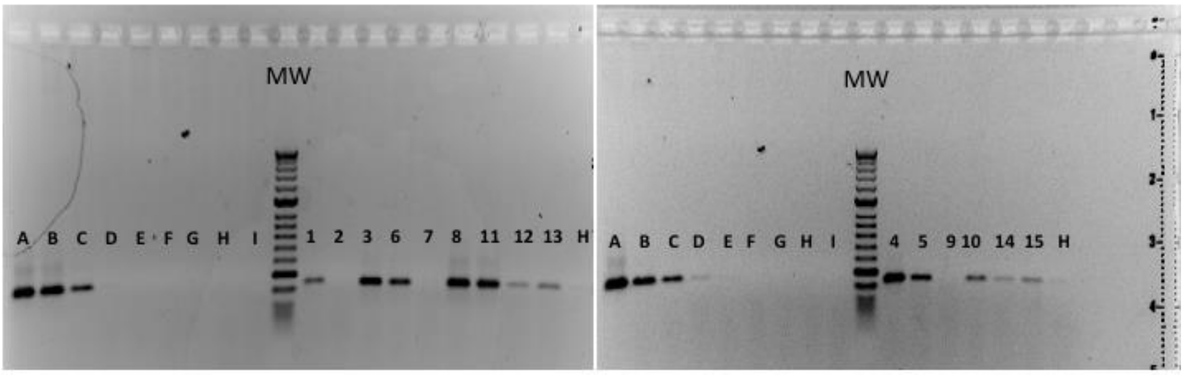


A= 10 000 000 HT1080
B= 1 000 000 HT1080 + 9 mln P19
C= 100 000 HT1080 + 9 900 000 P19
D= 10 000 HT1080 + 9 990 000 P19
E= 1 000 HT1080 + 9 999 000 P19
F= 100 HT1080 + 9 999 900 P19
G= 10 HT1080 + 9 999 990 P19
H= 1 HT1080 + 9 999 999 P19
I= 10 000 000 P19
H= Healthy lung

**Figure S5.** Uncropped image of the 1% agarose gel from Figure 2C.

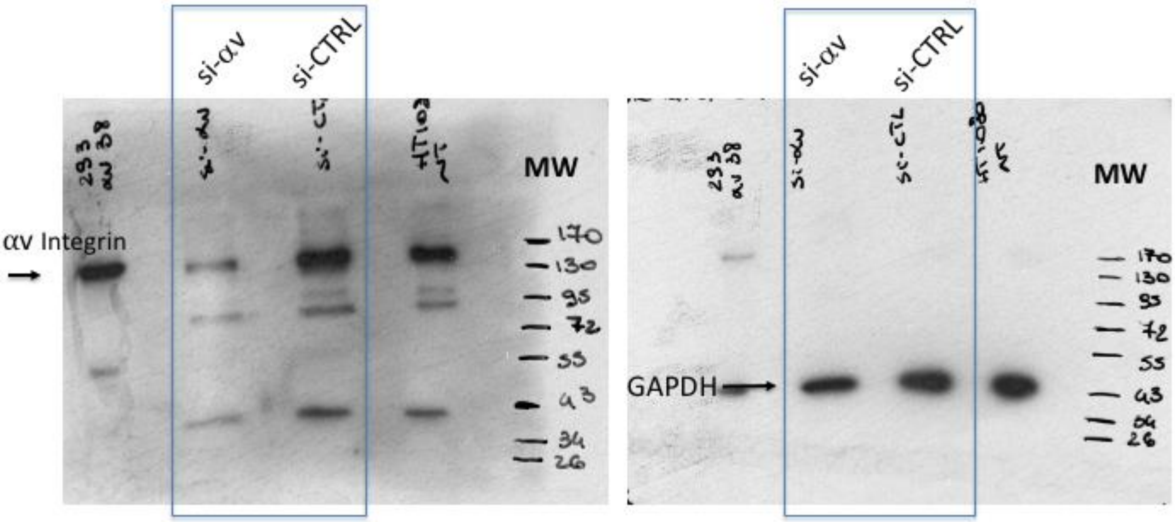


**Figure S6.** Whole blot image from Figure 4D.

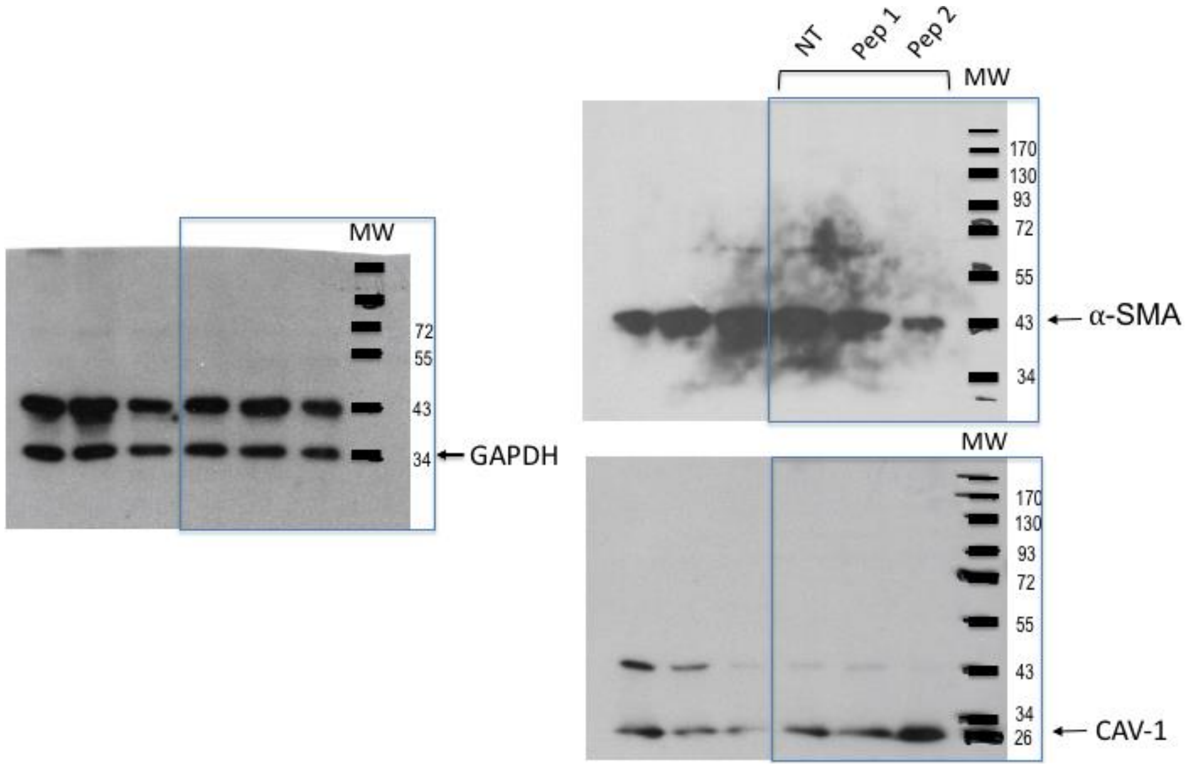


**Figure S7.** Whole blot image from Figure 5C.

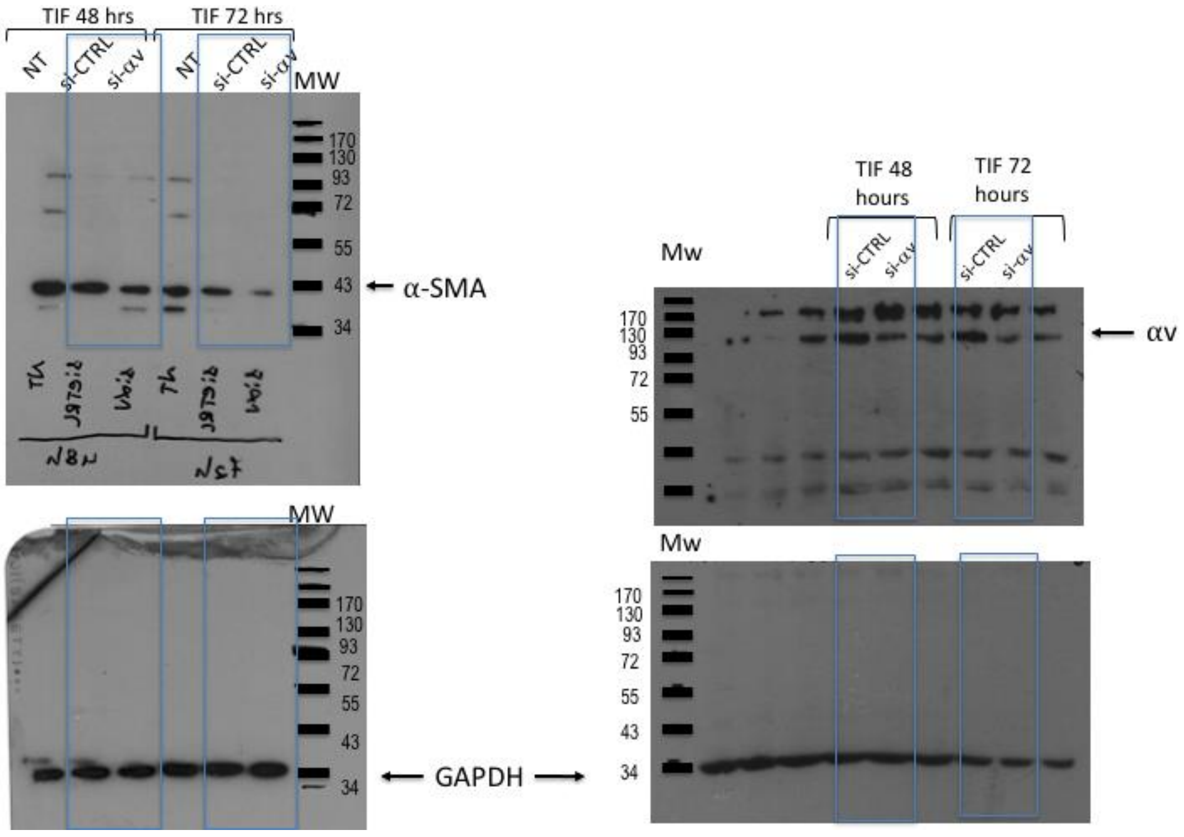


**Figure S8.** Whole blot image from Figure 6E.

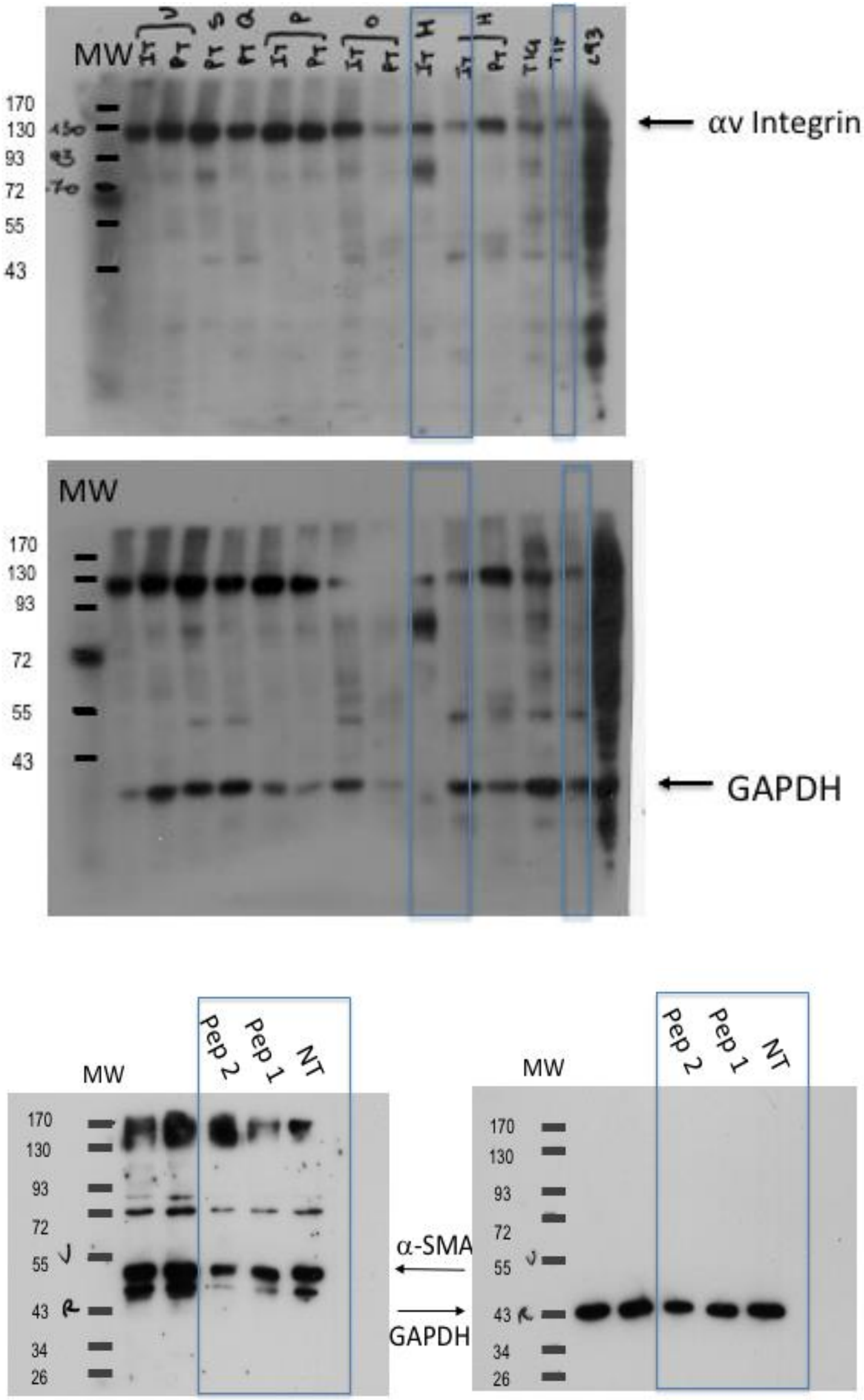


**Figure S9.** Whole blot image of Figure 7D,E.

**Table S1.** NMR resonance assignments [a] of Pep 1 in water solution at 10 °C.

| Residue | NH ($^3J_{\alpha N}$, −Δδ/ΔT)[b] | CαH | CβH | Others |
|---|---|---|---|---|
| Lys[136] | 8.39 (6.6, 8.7) | 4.58 | 1.72, 1.81 | 1.47(γ); 1.69(δ); 2.99(ε); 7.60 (ζ) |
| Pro[137] | | 4.44 | 2.32, 1.90 | 2.02(γ); 3.87, 3.65(δ) |
| Glu[138] | 8.71 (6.3, 7.3) | 4.29 | 2.04, 1.93 | 2.31(γ) |
| Ser[139] | 8.52 (7.4, 9.5) | 4.75 | 3.88, 3.75 | |
| Pro[140] | | 4.73 | 2.38, 1.94 | 2.05(γ); 3.85, 3.68 (δ) |
| Pro[141] | | 4.40 | 2.34, 1.92 | 2.05(γ); 3.84, 3.67(δ) |
| Glu[142] | 8.68 (5.9, 5.7) | 4.21 | 2.02, 1.94 | 2.31(γ) |
| Glu[143] | 8.55 (6.1, 6.2) | 4.26 | 2.03, 1.93 | 2.30(γ) |
| Leu[144] | 8.40 (7.2, 8.3) | 4.34 | 1.66 | 1.60(γ); 0.94, 0.88 (δ) |
| Lys[145] | 8.40 (7.2, 8.4) | 4.29 | 1.79, 1.86 | 1.42, 1.47 (γ); 1.68 (δ); 2.99 (ε) |

[a] Obtained at pH = 5.5, with TSP (δ 0.00 ppm) as reference shift. Chemical shifts are accurate to ± 0.02 ppm. [b] $^3J_{\alpha N}$ coupling constants in Hz−Δδ/ΔT = temperature coefficients (ppb/K) calculated in the range 10–25 °C. Further signals: $CH_3CO$, 2.01 ppm; $CONH_2$, 7.19, 7.61 ppm.

**Table S2.** NMR resonance assignments [a] of Pep 2 in water solution at 10 °C.

| Residue | NH ($^3J_{\alpha N}$, −Δδ/ΔT)[b] | CαH | CβH | Others |
|---|---|---|---|---|
| Lys[136] | 8.40 (6.9, 9.7) | 4.59 | 1.71, 1.81 | 1.49(γ); 1.71(δ); 2.99(ε); 7.60 (ζ) |
| Pro[137] | | 4.43 | 2.32, 1.88 | 2.03(γ); 3.88, 3.63(δ) |
| cGlu[138] | 8.53 (7.3, 9.4) | 4.29 | 2.07, 1.99 | 2.36, 2.44(γ) |
| Ser[139] | 8.36 (8.9, 9.1) | 4.52 | 3.60, 3.70 | |
| Pro[140] | | 5.06 | 2.08, 2.54 | 1.78, 1.97(γ); 3.56 (δ) |
| Pro[141] | | 4.37 | 2.44, 1.87 | 2.13(γ); 3.91, 3.62(δ) |
| Glu[142] | 8.90 (5.8, 4.8) | 4.24 | 2.06, 1.98 | 2.29(γ) |
| Glu[143] | 8.67 (5.8, 7.2) | 4.18 | 2.05, 1.94 | 2.25, 2.33(γ) |
| Leu[144] | 8.23 (7.1, 6.8) | 4.34 | 1.64 | 1.59(γ); 0.96, 0.89 (δ) |
| cLys[145] | 8.15 (7.4, 9.2) | 4.21 | 1.78, 1.82 | 1.40,1.49 (γ); 1.55 (δ); 3.08,3.38 (ε); 8.16 (ζ) |

[a] Obtained at pH = 5.5, with TSP (δ 0.00 ppm) as reference shift. Chemical shifts are accurate to ± 0.02 ppm. [b] $^3J_{\alpha N}$ coupling constants in Hz−Δδ/ΔT = temperature coefficients (ppb/K) calculated in the range 10−25 °C. Further signals: $CH_3CO$, 2.01 ppm; $CONH_2$, 7.14, 7.61 ppm.

**Table S3.** NOE derived upper limit constraints of Pep 1.

| Residue number | Residue type | Atom type | Residue number | Residue type | Atom type | Upper Limit Distance |
|---|---|---|---|---|---|---|
| 135 | ACE | QH | 136 | LYS | HN | 3.62 |
| 136 | LYS | HN | 136 | LYS | QB | 3.21 |
| 136 | LYS | HN | 136 | LYS | QG | 6.38 |
| 136 | LYS | HN | 137 | PRO | HD2 | 5.50 |
| 136 | LYS | HN | 137 | PRO | HD3 | 5.50 |
| 136 | LYS | HA | 137 | PRO | HD2 | 3.61 |
| 136 | LYS | HA | 137 | PRO | HD3 | 3.61 |
| 136 | LYS | HA | 137 | PRO | QD | 3.00 |
| 137 | PRO | HA | 138 | GLU | HN | 2.49 |
| 137 | PRO | HA | 139 | SER | HN | 4.97 |
| 137 | PRO | HD2 | 138 | GLU | HN | 5.50 |
| 137 | PRO | HD3 | 138 | GLU | HN | 5.50 |
| 138 | GLU | HN | 138 | GLU | HA | 2.86 |
| 138 | GLU | HN | 138 | GLU | QB | 3.21 |
| 138 | GLU | HN | 138 | GLU | QG | 5.82 |
| 138 | GLU | HA | 139 | SER | HN | 3.44 |
| 139 | SER | HN | 139 | SER | HB2 | 3.39 |
| 139 | SER | HN | 139 | SER | HB3 | 3.39 |
| 139 | SER | HN | 139 | SER | QB | 3.14 |
| 139 | SER | HN | 140 | PRO | QD | 5.34 |

**Table S3.** *Cont.*

| Residue number | Residue type | Atom type | Residue number | Residue type | Atom type | Upper Limit Distance |
|---|---|---|---|---|---|---|
| 139 | SER | HA | 140 | PRO | HD2 | 3.11 |
| 139 | SER | HA | 140 | PRO | HD3 | 3.11 |
| 139 | SER | HA | 140 | PRO | QD | 2.67 |
| 140 | PRO | HA | 141 | PRO | HD3 | 2.99 |
| 140 | PRO | HA | 142 | GLU | HN | 5.13 |
| 141 | PRO | HA | 142 | GLU | HN | 3.36 |
| 141 | PRO | HA | 143 | GLU | HN | 3.00 |
| 141 | PRO | HD2 | 142 | GLU | HN | 3.00 |
| 141 | PRO | HD3 | 142 | GLU | HN | 5.50 |
| 142 | GLU | HN | 142 | GLU | HA | 2.83 |
| 142 | GLU | HN | 142 | GLU | QB | 3.50 |
| 142 | GLU | HN | 142 | GLU | QG | 5.42 |
| 142 | GLU | HA | 143 | GLU | HN | 3.25 |
| 143 | GLU | HN | 143 | GLU | QB | 3.12 |
| 143 | GLU | HN | 143 | GLU | QG | 6.25 |
| 143 | GLU | HA | 144 | LEU | HN | 2.52 |
| 143 | GLU | HB2 | 144 | LEU | HN | 4.01 |
| 143 | GLU | HB3 | 144 | LEU | HN | 4.01 |
| 143 | GLU | QB | 144 | LEU | HN | 3.49 |
| 143 | GLU | QG | 144 | LEU | HN | 6.38 |
| 144 | LEU | HN | 144 | LEU | QB | 4.18 |
| 144 | LEU | HN | 144 | LEU | HG | 4.19 |
| 144 | LEU | HN | 144 | LEU | QD1 | 6.53 |
| 144 | LEU | HN | 144 | LEU | QD2 | 6.53 |
| 144 | LEU | HA | 144 | LEU | QD1 | 6.53 |
| 144 | LEU | HA | 144 | LEU | QD2 | 6.53 |
| 145 | LYS | HN | 145 | LYS | HB2 | 3.95 |
| 145 | LYS | HN | 145 | LYS | HB3 | 3.95 |
| 145 | LYS | HN | 145 | LYS | QB | 3.18 |
| 145 | LYS | HN | 145 | LYS | HG2 | 5.50 |
| 145 | LYS | HN | 145 | LYS | HG3 | 5.50 |
| 145 | LYS | HA | 145 | LYS | QD | 6.38 |
